# Cardiac Myosin-Binding Protein C in Suspected Acute Coronary Syndrome: From Sarcomeric Injury Biology to Decision-Grade Risk Stratification

**DOI:** 10.3390/ijms27156934

**Published:** 2026-08-02

**Authors:** Michal Pruc, Maciej Maslyk, Milosz J. Jaguszewski, Lukasz Szarpak

**Affiliations:** 1 Institute of Medical Sciences, The John Paul II Catholic University of Lublin, 20-708 Lublin, Poland; 2 Institute of Biological Sciences, The John Paul II Catholic University of Lublin, 20-708 Lublin, Poland; 3 First Department of Cardiology, Medical University of Gdansk, 80-214 Gdansk, Poland; 4 Henry JN Taub Department of Emergency Medicine, Baylor College of Medicine, Houston, TX 77030, USA

**Keywords:** cardiac myosin-binding protein C, cMyBP-C, cMyC, MYBPC3, acute coronary syndrome, myocardial infarction, high-sensitivity cardiac troponin assays, emergency department triage, point-of-care testing, microvascular obstruction

## Abstract

Cardiac myosin-binding protein C (cMyBP-C) is a cardiac-restricted sarcomeric protein; after cardiomyocyte injury, circulating intact cMyBP-C and/or cMyBP-C fragments, collectively referred to here as the cMyC biomarker signal, appear rapidly in blood. In suspected acute coronary syndrome (ACS), its most important potential role is not as another marker of injury but as a decision-enhancing biomarker beyond symptoms, electrocardiography, cardiac troponin T and I concentrations measured with high-sensitivity assays (hs-cTnT and hs-cTnI), time from pain onset, and pre-test probability. This narrative review separates three clinical tasks frequently conflated in the biomarker literature: diagnosis of acute myocardial infarction, emergency-department triage, and prediction of short-term or post-infarction risk. We integrate cMyBP-C sarcomeric architecture, N-terminal regulatory biology, phosphorylation, proteolysis, circulating fragments, assay epitopes, analytical stability, diagnostic algorithms, point-of-care testing, ST-segment elevation myocardial infarction reperfusion biology, and major confounders including renal dysfunction, heart failure, age, sex, and chronic ventricular remodeling. Current evidence supports further evaluation of cMyC as an adjunct in early presenters and accelerated diagnostic pathways. However, diagnostic safety and efficacy have not been consistently reproduced across platforms and populations, and external validation—particularly of rule-out performance—remains insufficient for routine clinical use. Recurrent injury assessment and post-infarction risk phenotyping remain promising but incompletely validated applications. Before guideline adoption, cMyC needs phenotype-specific, multicenter implementation trials demonstrating incremental net benefit, cost-effectiveness, and patient-level safety compared with contemporary hs-cTnT- and hs-cTnI-based clinical decision algorithms.

## 1. Introduction

High-sensitivity cardiac troponin (hs-cTn) assays have transformed the evaluation of patients with suspected acute coronary syndrome (ACS) [1]. These assays made small amounts of myocardial injury measurable, enabled accelerated 0/1 h and 0/2 h clinical decision pathways for the rule-out and rule-in of myocardial infarction, and reduced the historical dependence on prolonged observation [1,2]. Yet the clinical problem has not disappeared. Emergency physicians and cardiologists still face patients who arrive within the first hour of pain, patients with borderline biomarker elevations, older individuals with chronic myocardial injury, patients with chronic kidney disease (CKD) or heart failure (HF), and patients with recurrent chest pain shortly after an index infarction [3,4,5]. In these settings, the principal problem is not the absence of a biomarker but the uncertainty associated with biomarker interpretation [3,4,5]. Contemporary hs-cTn algorithms address much of this uncertainty but remain dependent on assay-specific concentrations, serial changes, and clinical context [6].

Cardiac myosin-binding protein C (cMyBP-C) is a cardiac-restricted and abundant sarcomeric protein. Following cardiomyocyte injury, circulating intact protein and/or cMyBP-C fragments measured as cMyC appear rapidly in blood, supporting its evaluation as a complementary biomarker of myocardial injury [7,8,9,10]. That rationale is compelling, but it is insufficient for a mature review. A useful biomarker review for contemporary ACS must ask a stricter question: does cMyC alter a decision that would otherwise have been made using clinical history, electrocardiography (ECG), and cardiac troponin T and I concentrations measured using high-sensitivity assays (hs-cTnT and hs-cTnI, respectively), symptom timing, and pre-test probability [11,12]? The relevant decisions are concrete: discharge versus observation, repeat sampling versus single-sample rule-out, early invasive evaluation versus non-invasive work-up, intensified follow-up versus routine care, and whether a high post-infarction risk phenotype has been identified [11,13,14].

This narrative review therefore separates three domains often compressed into one: diagnosis, triage, and prediction. Diagnosis is the identification of acute myocardial infarction (AMI), especially non-ST-segment elevation myocardial infarction (NSTEMI), among patients with possible ACS [7,11]. Triage is the operational task of safely allocating patients to rule-out, observation, or rule-in pathways early enough to change emergency department (ED) flow [11,13]. Prediction is broader: 30-day major adverse cardiovascular events (MACE), mortality, recurrent infarction, urgent revascularization, acute HF, infarct size, microvascular obstruction, left ventricular remodeling, and length of stay [14,15]. The distinction matters because a marker can be diagnostically accurate without being operationally useful, and it can predict risk without improving management. Accordingly, this review adopts a translational molecular-medicine perspective, linking the sarcomeric biology and release kinetics of cMyBP-C with assay characteristics and clinically relevant diagnostic, triage, and prognostic decisions [8,11,15,16]. The review is intentionally critical. It does not argue that cMyC should replace troponin. It argues that cMyC may be valuable where current high-sensitivity cardiac troponin (hs-cTn) clinical decision pathways leave residual decision uncertainty, and that this hypothesis now requires endpoint-driven implementation science.

## 2. Methods

This narrative review used a targeted literature-selection strategy designed to support clinical interpretation rather than formal meta-analysis. PubMed/MEDLINE was searched from database inception to 1 June 2026 using combinations of the terms “cardiac myosin-binding protein C”, “cMyBP-C”, “cMyC”, “MYBPC3”, “acute coronary syndrome”, “myocardial infarction”, “ST-segment elevation myocardial infarction”, “STEMI”, “non-ST-segment elevation myocardial infarction”, “NSTEMI”, and “microvascular obstruction”. Priority was given to prospective diagnostic cohorts, algorithm-derivation and validation studies, studies comparing cMyC directly with hs-cTnT and/or hs-cTnI, point-of-care studies, and studies linking cMyC to imaging or clinical outcomes.

Mechanistic and molecular studies were included when they explained protein structure, phosphorylation, proteolysis, fragment release, assay epitopes, or the biological interpretation of circulating cMyC. Reference lists of key papers were reviewed to identify additional foundational work. Reviews were used to contextualize the field but were not treated as primary evidence for diagnostic or prognostic performance. Where possible, study-level data were extracted, including cohort size, endpoint definition, symptom-onset and blood-sampling windows, assay platform, sample matrix, area under the curve (AUC), sensitivity, specificity, negative predictive value, positive predictive value, reported thresholds, and rule-out/rule-in/observe proportions. When a metric was not reported or could not be unambiguously extracted from the article record, it was classified as not reported (NR) rather than imputed. The review did not pool estimates because the studies differ in assay platform, clinical population, symptom-onset distribution, adjudication method, comparator troponin assay, and intended use case.

Because this was a narrative review, a formal Grading of Recommendations Assessment, Development and Evaluation (GRADE) certainty rating was not applied. Instead, interpretive confidence was assessed using a structured study-level framework defined for this review. The framework considered prospective versus retrospective design; multicenter recruitment or external validation; prespecification of thresholds; completeness of reporting for the area under the receiver-operating-characteristic curve, sensitivity, specificity, negative predictive value, and positive predictive value; explicit definition of symptom-onset and blood-sampling windows; blinded clinical endpoint adjudication; and adequate description of the assay platform, analytical performance, antibody epitopes, and sample matrix. Interpretive confidence was classified as high when a study was prospective, included multicenter recruitment or external validation, used prespecified thresholds, reported the principal diagnostic metrics and sampling windows, and adequately described the assay. It was classified as moderate when one or two of these elements were incomplete but the study remained interpretable for its intended context, and as limited when major diagnostic metrics or sampling windows were missing, thresholds were predominantly post hoc, assay characterization was insufficient, or the design did not permit transfer to the proposed clinical use. For non-diagnostic analytical or mechanistic studies, confidence was restricted to the directly reported analytical or mechanistic claim and did not imply clinical validation. Missing key metrics were treated as limitations of the affected comparison, and studies with limited interpretive confidence were not used to support direct cross-study claims of superiority.

The designation not reported (NR) was not interpreted as a negative result and was not imputed. When a study did not report a key metric, that metric was excluded from the relevant comparison and the resulting limitation was recorded in the study-level evidence matrix. Because assay platforms, clinical populations, renal phenotypes, and sampling schedules differed materially, the qualitative synthesis was additionally stratified, where data permitted, by time from symptom onset (<1 h, 1–3 h, 3–6 h, and >6 h or late presentation), renal function (preserved renal function, chronic kidney disease, and hemodialysis), and assay platform or epitope configuration. The study-level evidence matrix and the assay/threshold summary are provided in Supplementary Tables S1 and S2, respectively.

## 3. Molecular Architecture of MYBPC3-Encoded cMyBP-C

cMyBP-C is an approximately 140-kDa thick-filament-associated protein encoded by the myosin-binding protein C3 gene (MYBPC3) and localized within the C-zone of the cardiac sarcomere [16,17]. It contains a modular series of immunoglobulin-like and fibronectin type III domains designated C0 through C10. The C-terminal region contributes to thick-filament anchoring, whereas the regulatory N-terminal C0-C2/M-domain region interacts with myosin and actin and tunes cross-bridge recruitment [16,17,18]. Experimental evidence also indicates that cMyBP-C can interact with components of the cardiac troponin complex, including cardiac troponin T and cardiac troponin I, in a calcium- and phosphorylation-dependent manner [19].

Phosphorylation is central to the molecular interpretation of cMyBP-C biology. Phosphorylation modulates cMyBP-C interactions with myosin, actin, and the cardiac troponin complex and contributes to the physiological regulation of cross-bridge kinetics [16,18,19]. Dephosphorylation during ischemia–reperfusion, oxidative stress, or energetic failure may increase susceptibility to proteolysis, particularly in N-terminal regions relevant to circulating fragments [9,16]. Accordingly, circulating cMyC concentrations may reflect a composite of cardiomyocyte injury, sarcomeric protein abundance, phosphorylation-dependent susceptibility to proteolysis, the molecular forms recognized by the assay, and subsequent clearance. The relative contribution of each of these processes in human ACS remains incompletely characterized.

The distinction between intact protein and circulating fragments is important for assay interpretation. Immunoassays detect specific epitopes rather than an abstract, uniform molecular entity. Consequently, the measured cMyC concentration depends on antibody selection, epitope accessibility, calibrator design, matrix effects, and recognition of intact versus fragmented circulating forms [9,10]. For translational studies, the same numerical concentration may have different biological meaning if an assay preferentially recognizes intact cMyBP-C, specific N-terminal fragments, or a mixed pool of injury-related circulating species [9,10].

The clinical performance of cMyC may therefore depend not only on total circulating concentration but also on which molecular species the assay captures [9,10]. Future diagnostic and prognostic studies should report antibody epitopes, calibrator design, fragment recognition, sample matrix, and whether the assay preferentially detects intact cMyBP-C, N-terminal fragments, or mixed circulating forms [9,10]. Without this information, apparently similar cMyC thresholds may not be biologically or clinically interchangeable across platforms.

Most clinical studies have not directly separated intact cMyBP-C from individual circulating fragments. Assays using N-terminal antibody pairs may recognize intact protein together with multiple N-terminal-containing fragments and should not be described as measuring a single fragment isoform unless molecular-species specificity has been directly demonstrated. Reported reference limits and clinical cut-offs are therefore summarized by platform and epitope configuration in Supplementary Table S2 and should not be transferred across assays.

Clearance biology remains insufficiently resolved. Renal function and ongoing myocardial injury are associated with circulating cMyC concentrations, whereas the contributions of fragment size, protein binding, and non-renal clearance pathways remain insufficiently characterized [15,16]. This is clinically relevant because the populations in which biomarkers are most needed—older patients and patients with CKD, HF, ventricular hypertrophy, atrial remodeling, or diabetes—are also those in whom chronic myocardial injury and altered clearance are common [15,20,21]. A molecular-to-clinical review must therefore treat cMyC as an injury signal embedded in biological context, not as a lesion-specific marker of coronary thrombosis [11,22].

## 4. Nomenclature and Clinical Language: cMyBP-C, cMyC, and MYC

In this narrative review, cMyBP-C refers to the sarcomeric protein, whereas cMyC refers to the circulating biomarker signal or assay result. The distinction is retained because cMyC can be confused with the MYC proto-oncogene and its encoded c-Myc protein (MYC/c-Myc) in molecular oncology. The term cMyC is used only when discussing the circulating biomarker or assay result, and cMyBP-C is used when discussing structure, MYBPC3 biology, phosphorylation, proteolysis, or sarcomeric function [7,16].

This nomenclature matters clinically. When a proposed ED pathway uses a cMyC concentration, it is not reporting coronary thrombosis. It is reporting a cardiac sarcomeric injury signal [9,16,22]. The diagnostic meaning emerges only after integration with symptoms, electrocardiogram (ECG), hs-cTnT or hs-cTnI, time from pain onset, and pre-test probability [11,23]. These nomenclature distinctions are clinically relevant because cMyC, hs-cTnT, and hs-cTnI results are not interchangeable measures of myocardial injury. The underlying proteins and assays differ in molecular source, regulatory biology, circulating molecular forms, analytical behavior, and expected clinical use. Table 1 summarizes these differences and frames why cMyC should be evaluated as a potential adjunct or subgroup tool rather than as a generic replacement for established hs-cTnT- and hs-cTnI-based clinical decision pathways.

**Table 1:** Molecular, analytical, and clinical comparison of cMyBP-C/cMyC, cTnT/hs-cTnT, and cTnI/hs-cTnI.

Dimension	cMyBP-C/cMyC	cTnT/hs-cTnT	cTnI/hs-cTnI	Comparative Clinical Implication
Gene, molecular source, and sarcomeric role	MYBPC3-encoded, approximately 140-kDa C-zone thick-filament protein with C0–C10 domains; C-terminal anchoring and N-terminal regulatory functions [16,17].	TNNT2-encoded tropomyosin-binding subunit of the heterotrimeric cardiac troponin complex on the thin filament [24].	TNNI3-encoded inhibitory subunit of the cardiac troponin complex; suppresses actomyosin interaction at low Ca^2+^ [24].	All are cardiac sarcomeric proteins released during cardiomyocyte injury; none directly identifies coronary plaque rupture or thrombosis.
Regulatory biology	N-terminal interactions with myosin, actin, and components of the cardiac troponin complex, including cardiac troponin T and cardiac troponin I, may be calcium- and phosphorylation-dependent; phosphorylation also modulates cross-bridge recruitment and proteolytic susceptibility [16,18,19].	Anchors the troponin complex to tropomyosin and transmits Ca^2+^-dependent regulatory changes along the thin filament [24]. cTnT is a phosphoprotein whose phosphorylation state can modify thin-filament regulation and myofilament function [25].	Controls thin-filament inhibition and myofilament Ca^2+^ sensitivity; phosphorylation and dephosphorylation modulate relaxation and contractility [26].	The proposed early cMyC signal is supported by myocardial abundance and observed release kinetics, not by regulatory biology alone [7,8].
Circulating analyte detected by the assay	Assay-dependent mixture of intact cMyBP-C and injury-related fragments; the result depends on antibody epitopes and calibrator design [9,10].	Assay-dependent circulating intact, complexed, and fragmented cTnT forms [27].	Assay-dependent circulating free, complexed, and fragmented cTnI forms [27].	Results are not molecularly interchangeable; circulating-form heterogeneity may influence kinetics and thresholds.
Established or proposed clinical strength	High myocardial abundance and rapid release; potential value in very early presenters, accelerated triage, and observe-zone reduction [7,8,9,11]. Faster kinetics provide a rationale, but not yet decision-grade evidence, for use in suspected reinfarction [8].	Extensively validated assay-specific algorithms and guideline-established clinical use [2,3,4,28].	Extensively validated platform-specific algorithms and guideline-established clinical use [2,3,4,28].	cMyC must demonstrate incremental decision benefit beyond contemporary hs-cTnT and hs-cTnI clinical decision pathways.
Major interpretive limitations	Age, renal dysfunction, HF, LV remodeling, chronic myocardial injury, incomplete assay harmonization, and incompletely validated demographic thresholds [15,20,21,29].	Age, CKD, HF, structural heart disease, chronic myocardial injury, and assay-specific thresholds [3,4].	Age, CKD, HF, structural heart disease, chronic myocardial injury, and platform-specific thresholds [3,4].	No result alone distinguishes type 1 MI from type 2 MI or acute non-ischemic myocardial injury [22].
Implementation readiness	Investigational; requires platform harmonization, reference-limit studies, POCT validation, and prospective decision-embedded trials.	Guideline-embedded and widely implemented.	Guideline-embedded and widely implemented.	Routine cMyC adoption requires additional safety, clinical-utility, workflow, and cost-effectiveness evidence.
Potential combined strategy	Potentially earlier and more dynamic sarcomeric injury signal [7,8,11,13,30].	Guideline-established, assay-specific rule-out and rule-in pathway retained as the clinical backbone [2,3,4,28].	Guideline-established, platform-specific rule-out and rule-in pathway retained as the clinical backbone [2,3,4,28].	A dual-marker strategy may exploit complementary kinetics while preserving established hs-cTn pathways, but it requires prespecified handling of concordant and discordant results and prospective demonstration of incremental safety and clinical utility [11,13,30]. Operational evidence is summarized in Table 2.

Abbreviations: CKD, chronic kidney disease; cMyBP-C, cardiac myosin-binding protein C; cMyC, circulating cMyBP-C biomarker signal; cTnI, cardiac troponin I; cTnT, cardiac troponin T; HF, heart failure; hs-cTnI, cardiac troponin I measured with a high-sensitivity assay; hs-cTnT, cardiac troponin T measured with a high-sensitivity assay; kDa, kilodalton; LV, left ventricular; MI, myocardial infarction; MYBPC3, myosin-binding protein C3 gene; POCT, point-of-care testing; TNNI3, troponin I3, cardiac type gene; TNNT2, troponin T2, cardiac type gene.

**Table 2:** Decision algorithms and operational outputs relevant to cMyC implementation.

Strategy	Decision Metric	Numerical Data Available	Primary Clinical Promise	Main Limitation
Single-sample cMyC	Immediate rule-out or rule-in at presentation	0 h triage 48.1% in APACE comparison vs. 21.2% for ESC hs-cTnT and 29.9% for ESC hs-cTnI [30]	Operationally attractive in ED crowding and early presenters	Safety depends on exact assay, symptom timing, and low-concentration precision
cMyC 0/1 h algorithm	0 h concentration + 1 h delta	APACE validation: rule-out 52.3%, rule-in 18.4%, observe 29.3%; sensitivity 99.1% (95% CI 97.0–100), NPV 99.6% (95% CI 98.9–100), specificity 93.6% (95% CI 91.4–95.6), PPV 71.1% (95% CI 63.1–79.0) [30]. Japanese external validation: cMyC rule-out 56.5%, NPV 95.2%, sensitivity 83.3%; rule-in 19.2%, PPV 70.0%, specificity 90.3%; hs-cTnT rule-out NPV and sensitivity 100% [29].	Algorithmic evidence with discordant external rule-out safety	Rule-out safety was not reproduced across platforms and populations; cMyC is not suitable for stand-alone discharge without further validation.
Dual marker: cMyC + hs-cTnT and/or hs-cTnI	Combined early injury signals	Lopez-Ayala 2025: triage efficacy 60.0% vs. 26.8% with hs-cTnT alone without compromising safety endpoints [11]	Most credible near-term implementation model	Discordance may increase complexity unless pathways are pre-specified
Dual-marker strategies involving cMyC or copeptin	Injury plus systemic stress physiology	Across the evaluated dual-marker strategies, NPV ranged from 99.1% to 100% and sensitivity from 96.2% to 100%; the combinations highlighted by the authors included hs-cTnT plus cMyC and hs-cTnT plus copeptin [13].	Potential support for rapid rule-out strategies combining an injury marker with either an additional sarcomeric injury marker or a systemic stress marker.	Copeptin specificity and interpretability remain concerns
cMyC + clinical score	Biomarker plus ECG/history/risk score	No definitive randomized implementation trial was identified in the literature search conducted for this review	Avoids biomarker-only discharge	Risk scores differ by setting; net benefit must be shown
Prehospital cMyC	Ambulance-level early biochemical triage	Prehospital AUC 0.839; sensitivity and NPV reached 100% at the 10 ng/L threshold after at least 2 h of symptoms in the reported cohort [31]	Warrants prospective evaluation for earlier biochemical triage and pathway routing	False reassurance in very early presenters would be unacceptable
STEMI serial cMyC	Infarct-size/MVO risk quantification	Serial CMR-linked studies emerging; diagnostic metrics not applicable [32]	Potential post-reperfusion risk phenotype	Diagnosis already established; must beat or complement hs-cTnI and CMR endpoints

Abbreviations: APACE, Advantageous Predictors of Acute Coronary Syndrome Evaluation; AUC, area under the receiver operating characteristic curve; CI, confidence interval; cMyC, circulating cMyBP-C biomarker signal; CMR, cardiac magnetic resonance; ECG, electrocardiogram; ED, emergency department; ESC, European Society of Cardiology; hs-cTnI, cardiac troponin I measured with a high-sensitivity assay; hs-cTnT, cardiac troponin T measured with a high-sensitivity assay; MVO, microvascular obstruction; NPV, negative predictive value; NSTEMI, non-ST-segment elevation myocardial infarction; PPV, positive predictive value; STEMI, ST-segment elevation myocardial infarction.

## 5. Analytical Readiness: Assay Epitopes, Stability, Thresholds, and Standardization

The translational gap for cMyC is not limited to clinical evidence. It also includes assay science. A clinically deployable cMyC-based clinical decision pathway requires a defined lower limit of detection, lower limit of quantification, precision across the clinical decision range, stability under sample-handling conditions compatible with routine emergency department (ED) workflows, reproducible calibration, and traceable reference intervals [10,11]. For a protein subject to proteolysis and fragment circulation, antibody epitope selection is not a technical footnote but a biological determinant of what the test is measuring [9,16].

A reference 99th-percentile concentration can identify values above the distribution observed in an apparently healthy population, but it should not automatically be interpreted as an optimized rule-out or rule-in threshold for suspected ACS [23,29]. Rule-out and rule-in algorithms require context-of-use thresholds optimized for safety and efficacy. A low threshold may provide sensitivity and negative predictive value but at the cost of excessive observation. A high rule-in threshold may provide specificity and positive predictive value but risks missing smaller or earlier infarcts [11,13]. Delta criteria add temporal information but depend on the sampling interval and analytical imprecision at low concentrations [33].

**Figure 1:**
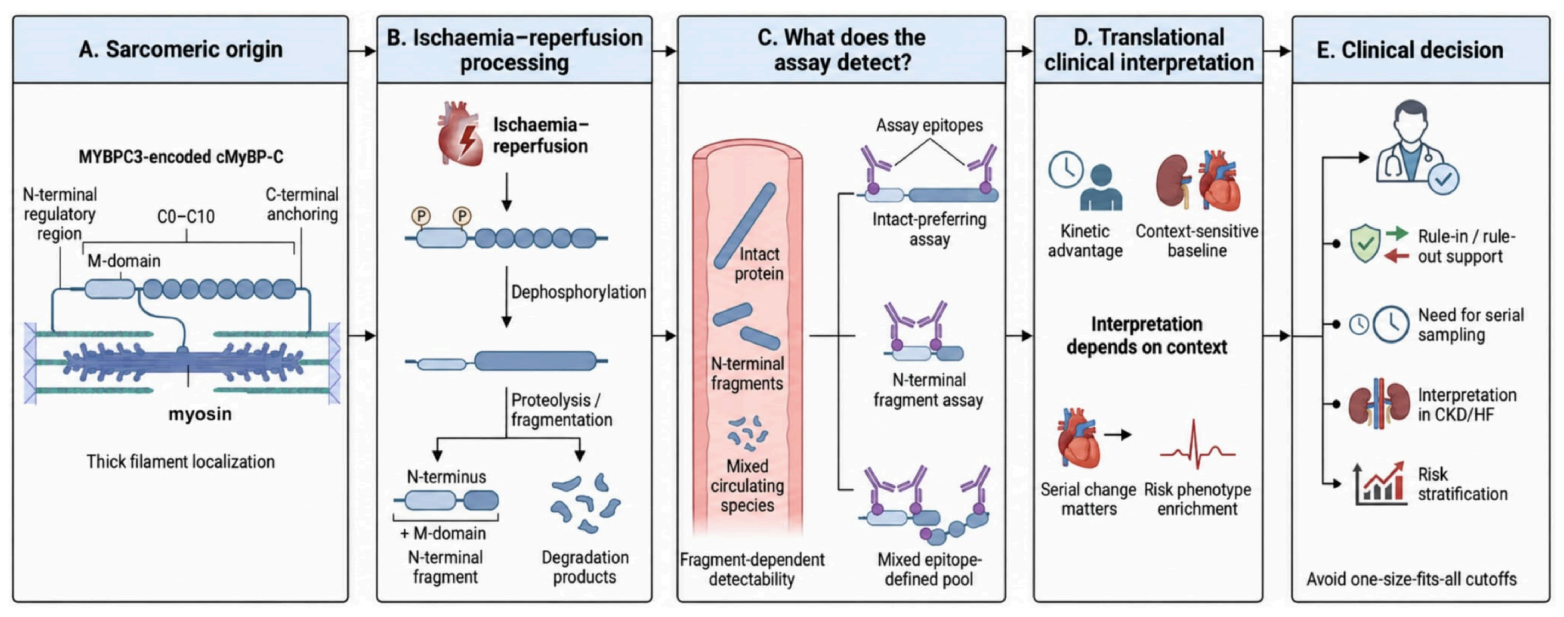
Molecular-to-clinical framework integrating the determinants of circulating cMyC with relevant clinical decisions. Note: The domain architecture is retained after dephosphorylation, whereas proteolytic fragmentation is shown as a separate subsequent process. The key message is that cMyC concentration is shaped by sarcomeric origin, ischemia–reperfusion processing, assay epitope recognition and patient phenotype; it should therefore be interpreted as decision-support information rather than as a stand-alone diagnostic label. The sarcomeric architecture and regulatory biology are supported by references [16,17,18,19]; ischemia–reperfusion-related processing and release kinetics by [7,8,9,16]; assay epitope and fragment recognition by [9,10]; phenotype-dependent interpretation by [15,20,21,29]; and the clinical decision framework by [11,12,13,14,23]. Throughout the figure, cMyBP-C denotes the intracellular sarcomeric protein, whereas cMyC denotes the circulating biomarker signal measured by an assay; neither term refers to the MYC proto-oncogene or c-Myc protein. The pale-red compartment in panel (**C**) represents the circulating blood compartment and does not indicate a separate mechanistic or evidentiary category.

External transportability is not guaranteed. Sex-specific and population-specific reference limits, assay platform effects, renal function, age, and comorbidity may shift the distribution of cMyC [15,20,29]. This is where molecular medicine meets implementation: a sarcomeric protein can be biologically elegant but clinically unsafe if cut-offs are transferred across platforms or populations without validation [10,29]. The molecular and analytical issues described above are not technical details detached from clinical practice. They determine what a circulating cMyC value can reasonably mean in suspected ACS. Figure 1 summarizes the molecular-to-clinical framework used throughout this review: MYBPC3-encoded sarcomeric origin, ischemia–reperfusion-related dephosphorylation and proteolysis, assay-dependent recognition of intact protein and fragments, and context-sensitive clinical interpretation. This framework explains why cMyC should not be evaluated as a generic myocardial infarction marker. Its clinical value depends on the decision task: early rule-out or rule-in support, observe-zone reduction, need for serial sampling, interpretation in chronic injury states such as CKD or HF, and risk stratification after myocardial infarction. The following sections therefore assess cMyC according to clinical phenotype and decision context rather than biomarker elevation alone.

Across studies, three sources of heterogeneity materially modified interpretation. First, the apparent diagnostic advantage of cMyC was greatest in the earliest symptom-onset strata and narrowed as hs-cTnT and hs-cTnI concentrations and deltas matured [7,11,30]. Second, CKD, hemodialysis, HF, and ventricular remodeling shifted baseline distributions and reduced the transferability of fixed thresholds [15,20,21,29]. Third, concentrations obtained with N-terminal epitope-pair assays, alternative automated platforms, enzyme-linked immunosorbent assay (ELISA)-based research methods, and investigational POCT systems could not be treated as interchangeable [9,10,14,29]. These strata were therefore summarized qualitatively rather than pooled, and cross-platform threshold transfer was avoided (Supplementary Tables S1 and S2).

## 6. Diagnostic Performance in Suspected ACS: cMyC Versus hs-cTnT and hs-cTnI

The relevant comparator is not generic troponin but hs-cTnT and hs-cTnI, considered separately. These assays differ in molecular target, analytical platform, circulating molecular forms, and assay-specific clinical thresholds [3,4,27]. The cMyC literature is strongest when it compares cMyC directly with both hs-cTnT and hs-cTnI and asks whether cMyC is an alternative, an adjunct, a subgroup tool, or an interesting marker without sufficient decision impact [7,11,34].

The 2017 direct comparison study established much of the clinical enthusiasm: cMyC was evaluated in unselected ED patients with symptoms suggestive of AMI and compared with high-sensitivity and standard troponin assays [7]. Subsequent studies refined the concept into algorithmic use. The 2022 Advantageous Predictors of Acute Coronary Syndrome Evaluation (APACE) 0/1 h study is particularly important because it moved from biomarker association to a decision pathway. In its validation cohort, the cMyC algorithm classified patients into rule-out, rule-in, and observe groups with very high negative predictive value for NSTEMI, and it achieved a larger proportion of immediate 0 h decisions than European Society of Cardiology (ESC) hs-cTnT and hs-cTnI algorithms [30].

This does not mean cMyC is ready to replace hs-cTn. The proper reading is narrower: cMyC may increase early triage efficacy in selected cohorts [11,13,30], but external validation has introduced important caution. In the Japanese cohort, the cMyC 0/1 h algorithm classified 43.8% of patients as rule-out at presentation and 56.5% after 1 h; however, the rule-out arm had a negative predictive value (NPV) of 95.2% and sensitivity of 83.3%. Rule-in occurred in 10.0% at 0 h and 19.2% after 1 h, with a positive predictive value (PPV) of 70.0% and specificity of 90.3%. By comparison, the hs-cTnT algorithm achieved rule-out NPV and sensitivity of 100% [29]. Thus, cMyC showed potentially useful rule-in performance, but cMyC-based rule-out safety was not reproduced in this external platform and population and is insufficient for stand-alone discharge.

## 7. Time from Symptom Onset: The Phenotype That May Decide Clinical Utility

**Figure 2:**
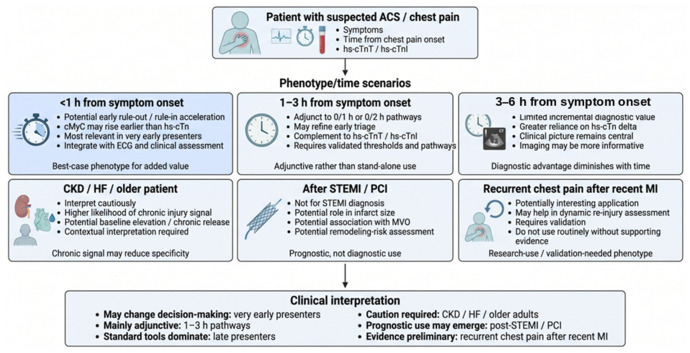
Proposed phenotype-specific use of cMyC in suspected acute coronary syndrome. Note: The potential clinical utility of cMyC depends on time from symptom onset and clinical phenotype. In very early presenters, cMyC may provide the greatest incremental value by accelerating rule-out or rule-in decisions when hs-cTn signals are still evolving. In patients presenting after 1–3 h, cMyC is more plausibly an adjunct to established 0/1 h or 0/2 h pathways. In later presenters, standard hs-cTn dynamics, clinical assessment and imaging are likely to dominate. In CKD, HF and older multimorbid patients, interpretation requires caution because chronic myocardial injury may reduce specificity. After STEMI/PCI, cMyC should be considered a potential prognostic or infarct-burden marker rather than a diagnostic tool. Recurrent chest pain after recent MI remains an attractive but insufficiently validated use case. Abbreviations: MVO, microvascular obstruction; PCI, percutaneous coronary intervention.

**Figure 3:**
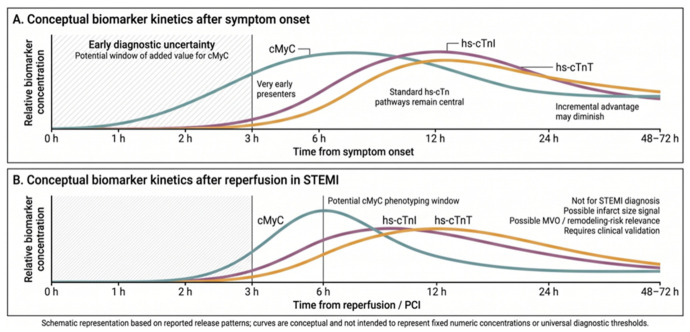
Conceptual biomarker kinetics of cMyC versus hs-cTnI and hs-cTnT after symptom onset and after reperfusion. Note: Panel (**A**) illustrates schematic biomarker release patterns after symptom onset in suspected ACS. cMyC may provide an earlier circulating signal during the first hours after injury, when diagnostic uncertainty is greatest and hs-cTnT and hs-cTnI concentrations or deltas may still be evolving. This potential advantage is expected to diminish as standard hs-cTn pathways become increasingly informative. Panel (**B**) illustrates conceptual kinetics after reperfusion in STEMI. In this setting, cMyC should not be viewed as a diagnostic marker for STEMI, but as a potential post-reperfusion phenotyping signal related to infarct burden, microvascular obstruction, and remodeling risk. The curves are schematic and were not fitted to or digitized from a single clinical cohort. Panel A is informed by reported early-release and diagnostic studies [7,8,9,11,30,31], whereas Panel B is informed by serial post-reperfusion and CMR-linked studies [32,35,36,37,38]. The figure illustrates relative temporal concepts rather than fixed concentrations, peak times, or universal diagnostic thresholds. Throughout the figure, cMyBP-C denotes the intracellular sarcomeric protein, whereas cMyC denotes the circulating biomarker signal measured by an assay; neither term refers to the MYC proto-oncogene or c-Myc protein.

The strongest potential advantage of cMyC is not in every chest-pain patient; it is in patients who present before the hs-cTnT or hs-cTnI concentration-time signal has fully evolved [7,11]. The time axis should be explicit: less than 1 h, 1–3 h, 3–6 h, and later presenters (>6 h), and recurrent pain after recent MI. The proposed added value of cMyC is fundamentally kinetic: it is expected to be greatest when MI is present but the hs-cTnT or hs-cTnI signal is still evolving. Figure 2 summarizes the main phenotype- and time-dependent scenarios in which cMyC may provide additional clinical value. Figure 3 provides a schematic representation of cMyC, hs-cTnI and hs-cTnT release patterns after symptom onset and after STEMI reperfusion, emphasizing the early diagnostic uncertainty window and the potential post-reperfusion phenotyping window. These curves should be interpreted conceptually rather than as fixed concentration-time profiles. Their purpose is to clarify where cMyC may plausibly add information: early diagnostic acceleration in suspected ACS and post-reperfusion injury phenotyping after STEMI, rather than universal replacement of established hs-cTn pathways.

In patients presenting within the first hour after symptom onset, an initially low hs-cTn concentration or an insufficiently developed serial change may not yet provide adequate biochemical confidence for rule-out. A single low troponin concentration may be reassuring only if symptom onset, assay, and clinical risk support that interpretation [23]. A rapidly released sarcomeric biomarker provides a biologically plausible advantage, but safe single-sample cMyC rule-out has not been established in patients presenting within 1 h. In the prehospital cohort, sensitivity and NPV reached 100% at the 10 ng/L threshold only after at least 2 h of symptoms [31]. For 1–3 h presenters, cMyC may still be useful but the incremental margin narrows as hs-cTn deltas become more informative [11]. At 3–6 h, the advantage is likely less diagnostic acceleration and more risk characterization or operational simplification.

Late presenters raise a different issue. If hs-cTn is already clearly positive and the diagnosis is clinically evident, cMyC may add little to diagnostic classification. Conversely, in late presenters with resolved symptoms, small infarcts, renal dysfunction, or chronic injury, cMyC could either clarify or complicate the picture depending on assay behavior [15,21]. These hypotheses need prospective subgroup analysis, not retrospective optimism.

Recurrent chest pain after recent MI is a particularly attractive but underdeveloped use case. A marker with faster clearance could potentially distinguish new injury from the tail of the prior event [8]. However, this setting is clinically and medico-legally unforgiving. It requires trials with adjudicated reinfarction, serial sampling, angiographic correlation where appropriate, and outcomes [8,9]. These scenarios distinguish settings in which cMyC may plausibly improve early decision-making from those requiring cautious or investigational interpretation. The central implication is that cMyC should not be treated as a uniform add-on test for all patients with chest pain. Its most plausible diagnostic value is concentrated in very early presenters and selected accelerated triage pathways, whereas chronic injury states, post-STEMI use, and recurrent pain after recent MI require phenotype-specific validation.

## 8. Single-Sample, 0/1 h, 0/2 h, and Dual-Marker Strategies

Modern ACS biomarker research is no longer about whether a marker is elevated. It is about how it performs inside a pathway. The clinically relevant pathways are single-sample rule-out, 0/1 h serial testing, 0/2 h serial testing, and dual-marker strategies [23,28,30]. Each addresses a different operational need. The practical question is not whether cMyC is elevated, but which diagnostic or triage strategy it improves. Table 2 summarizes the main cMyC-based operational approaches, the decision metric each strategy targets, the currently available numerical signal, and the principal limitation that must be addressed before implementation. Several points follow from this operational comparison. First, the strongest near-term rationale for cMyC lies in early triage efficiency and observe-zone reduction rather than in replacing hs-cTnT or hs-cTnI. Second, dual-marker strategies are attractive because they may combine complementary kinetics, but discordant results must be handled by prespecified pathways. Third, prehospital and point-of-care applications require particularly high safety standards because false reassurance in very early presenters would be clinically unacceptable.

Single-sample strategies are attractive in crowded EDs because they may reduce blood draws, serial laboratory testing, and time to disposition if cMyC replaces a repeat sample or shortens observation; adding cMyC to an otherwise unchanged hs-cTn pathway could instead increase laboratory workload and cost [12,23]. The cMyC 0 h signal in the APACE algorithm suggested that more patients could be classified immediately than with hs-cTnT or hs-cTnI algorithms [30]. That is potentially important, but single-sample safety must be judged by the lower confidence interval of sensitivity and negative predictive value, not by the point estimate alone. In high-throughput clinical environments, rare misses become visible.

The 0/1 h strategy is currently the most mature cMyC diagnostic framework, although external rule-out performance has been inconsistent [29,30]. It aligns with established ESC troponin logic by combining baseline concentration and absolute change [28]. The practical advantage is that clinicians understand this structure. The risk is that adding another marker could increase complexity unless it clearly reduces the observe zone or shortens the length of stay [11].

The dual-marker concept may be the most realistic near-term model. cMyC combined with hs-cTn can exploit complementary kinetics while preserving guideline familiarity [11,13]. Recent data suggest that cMyC can add incremental diagnostic value to hs-cTn and that dual-marker rule-out protocols may achieve high negative predictive values [11]. The proper endpoint for future trials should not be AUC alone. It should be decision reclassification, discharge rate, observation-zone reduction, 30-day event rate, patient satisfaction, cost, and clinician adherence [11,12].

## 9. Prognosis: From AMI Diagnosis to Post-Infarction Risk

Prediction must be broader than diagnosis. A biomarker that detects MI may also quantify injury burden, ventricular vulnerability, and risk of short-term complications [7,14,32]. For cMyC, the prognostic literature is growing but remains less definitive than the diagnostic literature. Beyond acute presentations, circulating cMyC has also been associated with disease severity and prognosis in stable coronary artery disease [39]. The 2026 point-of-care testing (POCT) study in AMI patients reported that admission cMyC was independently associated with 30-day major adverse cardiovascular events (MACE) after adjustment for clinical covariates, while explicitly noting that incremental predictive value requires further validation [14].

The distinction between independent association and incremental clinical utility is crucial. Many biomarkers remain statistically significant in multivariable models. Fewer improve calibration, discrimination, decision curves, and care pathways enough to justify routine testing [40]. For cMyC, the clinically meaningful question is whether admission concentration should change the intensity of monitoring, timing of echocardiography, threshold for invasive assessment, discharge timing, early HF surveillance, or follow-up interval [14,21].

A decision-grade prognostic study should therefore include predefined action thresholds, blinded outcome adjudication, contemporary treatment, competing biomarkers such as hs-cTn, N-terminal pro-B-type natriuretic peptide (NT-proBNP), renal function, and C-reactive protein (CRP), and metrics that clinicians can use [14,40]. Net reclassification improvement alone is not enough. A biomarker that moves patients between risk categories must point toward a management action [40].

## 10. STEMI, Reperfusion Injury, Infarct Size, and Microvascular Obstruction

Most cMyC discussions focus on suspected NSTEMI rule-out, but STEMI creates a different translational opportunity. In reperfused STEMI, the diagnosis is established from the clinical presentation and ECG, with angiography defining the coronary anatomy; therefore, the biomarker question is not primarily diagnostic. It is quantitative and prognostic: does cMyC reflect infarct size, microvascular obstruction, reperfusion injury, and risk of adverse remodeling [35,36]?

The 2026 STEMI cardiac magnetic resonance (CMR) study tested this question directly. Serial cMyC concentrations after primary percutaneous coronary intervention (PCI) correlated with acute and final infarct size by late gadolinium enhancement CMR, with the strongest relationship around 6 h after reperfusion [32]. This post-reperfusion concept is illustrated in Panel B of Figure 3, where the approximately 6 h window is presented as a potential cMyC phenotyping interval rather than a STEMI diagnostic use case. In this context, CMR-defined infarct size and microvascular obstruction should be treated as prognostic imaging phenotypes and validation endpoints for biomarker kinetics, rather than as evidence that cMyC alone can replace established troponin-based infarct-size assessment [35,36,37,38]. However, contemporary hs-cTnI and hs-cTnT measurements remain better-established biochemical comparators for infarct-size estimation and MVO prediction, especially at later time points [32,35,37]. This is a balanced and clinically important result: cMyC appears biologically informative after reperfusion, but current evidence does not support replacing established hs-cTnI or hs-cTnT measurements.

For future work, STEMI may be the best setting to connect molecular injury kinetics to imaging phenotypes and standardized CMR trial endpoints [36,37,38]. Trials should examine whether cMyC kinetics identify patients who need intensified HF prevention, repeat imaging, closer rhythm monitoring, or enrollment in cardioprotection studies [41,42]. The endpoint should not simply be correlation with CMR. It should be whether cMyC identifies a treatable risk phenotype.

## 11. Confounders: Renal Function, Age, Sex, Heart Failure, and Chronic Remodeling

No myocardial injury biomarker is clinically interpretable without its confounders. cMyC should not be described as a clean type 1 MI marker. General-population data show that circulating cMyC is measurable in most middle-aged individuals and is associated with left ventricular mass, left atrial volume, renal function, systolic and diastolic indices, and focal fibrosis [15]. Those associations make cMyC biologically interesting but diagnostically more complex.

Renal dysfunction is especially important. If cMyC concentrations are inversely associated with renal function, a fixed threshold may overcall acute injury in CKD, or at least increase the observe zone [3,15]. Whether cMyC performs better, worse, or differently than hs-cTnT and hs-cTnI in CKD cannot be assumed from aggregate ACS cohorts. It requires CKD-stratified validation with adjudicated type 1 MI, type 2 MI, acute non-ischemic injury, and chronic injury [3,43].

Age and sex also matter. Sex-specific thresholds are well established as a point of debate for hs-cTn, particularly hs-cTnI [3,44,45]. The Japanese cMyC reference work suggests that sex-specific interpretation may also be necessary for cMyC [29]. Older age, structural heart disease, atrial fibrillation, tachyarrhythmia, anemia, sepsis, chronic obstructive pulmonary disease exacerbation, and strenuous exercise could all produce myocardial injury without plaque rupture [3,22]. A mature review should explicitly state that cMyC may improve early injury detection while still requiring clinical adjudication of injury mechanism.

HF is not simply a confounder; it may be a separate use case. cMyC has been evaluated in acute HF, and concentrations may relate to myocardial injury burden and prognosis [21]. In suspected ACS, however, HF complicates the differential diagnosis and the biomarker interpretation [3]. The decision question becomes whether cMyC distinguishes ischemic from non-ischemic injury or merely measures risk. These are different claims.

Inherited cardiomyopathy and MYBPC3 variation. Pathogenic MYBPC3 variants are a major cause of hereditary hypertrophic cardiomyopathy and are associated with variable expression of ventricular hypertrophy and adverse cardiac remodeling [17,46,47]. These phenotypes constitute a biologically plausible potential confounder for circulating cMyC interpretation; however, direct evidence defining baseline cMyC distributions in genotype-positive individuals remains insufficient. Current evidence does not establish that genotype-positive individuals uniformly have persistently elevated circulating cMyC independently of phenotypic expression. Genotype-positive/phenotype-negative carriers should therefore not be assumed to have the same baseline distribution as patients with manifest hypertrophic cardiomyopathy. The prevalence of pathogenic MYBPC3 variants in unselected ED chest-pain cohorts and the diagnostic performance of cMyC in this subgroup have not been adequately reported. Genotype-adjusted thresholds therefore cannot currently be recommended. Future studies should separately evaluate genotype-positive/phenotype-negative carriers, genotype-positive patients with manifest cardiomyopathy, sarcomere-negative hypertrophic cardiomyopathy, and non-cardiomyopathy controls, with emphasis on baseline concentrations, serial changes, and discrimination of acute from chronic injury.

## 12. Where cMyC Is Unlikely to Help: Boundaries That Should Be Explicit

A high-quality review should be clear about where cMyC is not expected to solve the clinical problem. In late presenters with a convincing ischemic syndrome, diagnostic ECG evolution, and clearly positive hs-cTnT or hs-cTnI with an appropriate rise or fall, cMyC is unlikely to alter the diagnosis [22]. In that setting, the question is usually treatment strategy and anatomy, not whether MI exists. The APACE data confirm that the diagnostic advantage of cMyC over hs-cTnT was evident in early presenters but comparable in non-early presenters, reinforcing that the incremental value of cMyC concentrates in the first hours after symptom onset [11]. This boundary-setting is summarized in Table 3, which separates clinical phenotypes in which cMyC may plausibly add decision value from settings in which the signal may be diagnostically redundant, confounded by chronic injury, or insufficiently validated. The table also identifies the trial endpoint that would be needed to convert each proposed use case from biological plausibility into decision-grade evidence. The key point is that expected cMyC utility is not uniform across suspected ACS populations. Early presenters represent the strongest diagnostic-use case, whereas CKD, HF, late hs-cTn-positive NSTEMI, and suspected reinfarction require more cautious interpretation because cMyC may reflect chronic injury, infarct burden, delayed kinetics or unresolved adjudication rather than a new actionable diagnosis.

In CKD and HF, cMyC may be informative but interpretation is likely to be difficult [21,43]. These patients often have chronic myocardial injury, ventricular remodeling, altered clearance, and multiple competing causes of biomarker elevation. General-population data show that cMyC is inversely associated with renal function and positively associated with left ventricular mass and focal fibrosis [15]. In hemodialysis patients, cMyC exceeded the 99th percentile in 66% of cases compared with 99% for hs-cTnT, suggesting a potentially more favorable baseline profile, yet cMyC was not associated with mortality or cardiovascular events in that population [20].

For non-clinician readers, type 1 MI results from acute atherothrombotic coronary plaque disruption, whereas type 2 MI reflects an ischemic imbalance between myocardial oxygen supply and demand without acute coronary atherothrombosis. Type 3 MI denotes cardiac death with suspected acute myocardial ischemia before biomarker confirmation. Type 4 MI comprises PCI-related MI (type 4a), MI associated with stent or scaffold thrombosis (type 4b), and MI associated with restenosis (type 4c), whereas type 5 MI is related to coronary artery bypass grafting [22]. Acute non-ischemic myocardial injury is not classified as MI because evidence of acute myocardial ischemia is absent [22]. cMyC may improve injury detection or risk stratification, but it is unlikely to distinguish among these mechanisms without integration into phenotype-specific clinical algorithms.

In type 2 MI and acute non-ischemic myocardial injury, cMyC is unlikely to identify the trigger by itself [22]. Tachyarrhythmia, anemia, sepsis, hypoxemia, pulmonary embolism, hypertensive crisis, and postoperative stress can all injure the myocardium [3,22]. A more sensitive sarcomeric signal may increase detection without resolving etiology. This is clinically useful only if it changes management, surveillance, or prognosis rather than increasing diagnostic ambiguity.

In STEMI, cMyC should not be framed as a diagnostic tool. The diagnosis is established from the clinical presentation and ECG, often supported by angiographic findings; biomarker testing should not delay reperfusion [22]. The more credible question is whether serial cMyC quantifies infarct size, reperfusion injury, microvascular obstruction, or later ventricular remodeling [35]. For example, peak hs-cTnT has been shown to predict MVO with an AUC of 0.824 and clinically relevant MVO (>1.55% of LV mass) with an AUC of 0.837 [48].

## 13. Prehospital and Point-of-Care Applications

The prehospital setting is a natural target for cMyC because the value of earlier information increases when the patient is still outside the hospital [31]. Earlier prehospital cMyC information could be evaluated as an adjunct to routing or early cardiology-activation strategies. However, no outcome-based evidence currently supports cMyC-guided direct transport to PCI-capable centers. Earlier biochemical rule-out information could also be evaluated for its potential to reduce unnecessary ED congestion, although prehospital discharge decisions require a substantially higher evidentiary and regulatory threshold [12,31].

POCT changes the question from analytical performance alone to system performance [12]. For POCT deployment, diagnostic accuracy is necessary but insufficient; the decisive question is whether faster result availability changes routing, observation duration, or discharge decisions without increasing missed MI or downstream overtesting. From an implementation perspective, relevant safety requirements include turnaround time, device precision at low concentrations, operator training, quality control, sample type, electronic connectivity, and predefined failure modes. A central-laboratory test with excellent sensitivity but a 60-min result may be less useful than a slightly less precise POCT that is available before the second clinical assessment, provided safety is preserved.

The 2026 POCT study suggests that admission cMyC can be measured rapidly and may identify higher-risk patients after established AMI [14]. However, that study evaluated prognosis after confirmed AMI and does not validate POCT cMyC for diagnostic rule-out or discharge in suspected ACS. Diagnostic POCT and prognostic POCT are distinct use cases and require separate trials. A diagnostic POCT trial should compare cMyC-guided decisions with standard hs-cTn pathways in patients with suspected ACS, whereas a prognostic trial should test whether cMyC-guided management improves outcomes after confirmed AMI. Relevant endpoints include diagnostic safety, length of stay, downstream testing, missed MI, 30-day MACE, management change, and cost [11,12].

## 14. Implementation Science: From Promising Biomarker to Decision-Grade Evidence

A decision-grade biomarker must satisfy five conditions. First, it must be analytically robust across platforms and populations [10,29,33]. Second, it must improve diagnostic or prognostic performance beyond existing pathways [7,13]. Third, it must change a clinician’s decision [49,50]. Fourth, that changed decision must be safe [23,51]. Fifth, it must be operationally and economically acceptable [12,52].

**Figure 4:**
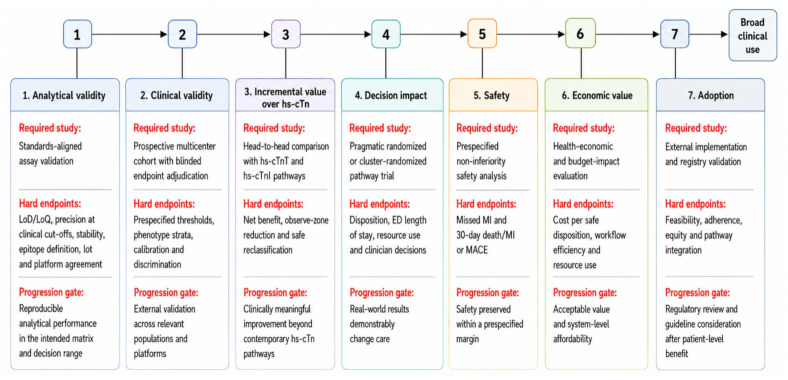
From biomarker to decision-grade evidence: an implementation framework for cMyC. Note: The clinical adoption of cMyC requires a staged evidentiary pathway extending beyond analytical detectability or diagnostic association. First, assays must demonstrate analytical validity, including precision, epitope and fragment standardization, analytical robustness and inter-assay consistency. Second, clinical validity must be established across multicenter populations and relevant phenotypes, including early presenters and patients with CKD, HF or multimorbidity. Third, cMyC must demonstrate incremental value over contemporary hs-cTnT and hs-cTnI clinical decision pathways, particularly through observe-zone reduction, earlier triage or phenotype-specific advantage. Fourth, biomarker results must change clinical decisions, including rule-in/rule-out allocation, emergency department length of stay, resource use or clinician decision support. Fifth, any accelerated pathway must preserve safety, including missed MI and 30-day MACE outcomes. Sixth, implementation must be economically justified. Finally, guideline adoption requires feasibility, clinician adherence and integration into validated care pathways. Each stage now specifies the required study design, hard endpoint, and progression gate; phenotype-specific designs and endpoints are detailed in Table 4.

The implementation pathway for cMyC should therefore be judged as a sequence from analytical validity to decision-grade clinical evidence, rather than as a simple progression from biomarker detectability to clinical adoption. Figure 4 summarizes the evidentiary steps required before cMyC can move from a promising sarcomeric injury signal to a broadly usable decision-support tool. This framework also reinforces why implementation should be phenotype-specific. A test may be analytically valid and clinically associated with myocardial injury, yet still fail to improve care if it does not reduce residual uncertainty, change disposition decisions, preserve safety, or justify its cost in the clinical pathway where it is deployed.

For cMyC, the most plausible implementation models are targeted rather than universal: early presenters, recurrent pain after recent MI, ambiguous hs-cTn results, prehospital triage, and selected high-risk AMI patients needing early prognostic stratification [11,31]. Universal testing of all chest-pain patients may dilute benefit and increase cost [12]. A phenotype-specific approach is more likely to demonstrate net clinical utility [14,49].

Patient safety, clinical governance, and clearly defined management of false-negative and discordant results are central to implementation. Rule-out pathways are judged harshly because false-negative events are rare but consequential [23,51]. Any cMyC pathway used for discharge must be tested with non-ischemic ECG, symptom timing, clinical risk, and outcome surveillance [23]. It must also define what clinicians should do with discordant results: low hs-cTn but high cMyC, high hs-cTn but low cMyC, and small deltas in either direction [13,34].

Cost-effectiveness should be analyzed from the beginning. A biomarker that adds a test cost may still be cost-saving if it reduces observation-zone occupancy, repeat sampling, unnecessary imaging, or admission [12,52]. But the economics will vary by health system, ED crowding, laboratory workflow, and payment model [12,52].

**Table 4:** Gaps-to-trials matrix for decision-grade evidence before guideline adoption.

Gap	Why It Matters	Design	Population	Primary Endpoint	Minimum Analytical Requirement
Incremental value beyond hs-cTnT and hs-cTnI separately	Clinicians use assay-specific troponin algorithms	Prospective multicenter diagnostic study with both hs-cTnT and hs-cTnI measured	Unselected suspected ACS	Net benefit and safe rule-out rate	Platform-specific LoD, LoQ, 99th percentile, delta imprecision
Early presenters	Most plausible biological use case	Enriched <3 h symptom-onset cohort	Chest pain <1 h and 1–3 h strata	30-day death/MI after discharge and observe-zone reduction	Low-end precision and symptom-time sensitivity analysis
Decision impact	AUC does not prove changed care	Cluster randomized ED implementation trial	ED chest-pain units	Length of stay, discharge rate, 30-day MACE	TAT compatible with real ED workflow
CKD/HF interpretation	Most difficult real-world population	Mechanism-adjudicated cohort	eGFR strata, acute/chronic HF, chronic injury	Specificity for type 1 MI and risk prediction	Renal and age-adjusted reference evaluation
Reinfarction	Troponin tail complicates care	Serial biomarker + imaging/angiography adjudication	Recent MI and recurrent chest pain	Recurrent MI diagnostic accuracy	Defined clearance and delta criteria
STEMI reperfusion biology	Diagnosis is easy; risk quantification is hard	Serial cMyC/hs-cTnI measurements combined with CMR and echocardiography	Reperfused STEMI	MVO, final infarct size, LV remodeling	Fragment/intact analyte characterization
POCT and prehospital deployment	Utility depends on turnaround time	Ambulance or ED pragmatic pathway trial	Paramedic and ED workflows	Time to decision, cost, safety	Whole-blood validation and stability
Global standardization	Cut-offs may not transfer	International reference-limit study	Well-characterized reference populations for upper reference limits, followed by independent suspected-ACS and disease cohorts for clinical threshold validation	Sex-, age-, and population-specific upper reference limits, followed by external validation of context-of-use rule-out, rule-in, and delta thresholds	Harmonized calibrators and epitope reporting
MYBPC3-related inherited cardiomyopathy	Genotype and expressed phenotype may alter baseline cMyC and the distinction between chronic and acute injury	Prospective genotype-informed diagnostic cohort	Genotype-positive/phenotype-negative carriers; genotype-positive manifest hypertrophic cardiomyopathy; genotype-negative hypertrophic cardiomyopathy; ACS controls	Baseline distributions, acute delta performance, and type 1 MI specificity	Platform-specific measurement with epitope and fragment characterization

Abbreviations: ACS, acute coronary syndrome; AUC, area under the receiver operating characteristic curve; CKD, chronic kidney disease; CMR, cardiac magnetic resonance; cMyC, circulating cMyBP-C biomarker signal; ED, emergency department; eGFR, estimated glomerular filtration rate; HF, heart failure; hs-cTnI, cardiac troponin I measured with a high-sensitivity assay; hs-cTnT, cardiac troponin T measured with a high-sensitivity assay; LoD, limit of detection; LoQ, limit of quantification; LV, left ventricular; MACE, major adverse cardiovascular events; MI, myocardial infarction; MVO, microvascular obstruction; POCT, point-of-care testing; STEMI, ST-segment elevation myocardial infarction; TAT, turnaround time.

## 15. Evidence Gaps and Trial Agenda

The evidence gap has shifted. It is no longer necessary to prove repeatedly that cMyC rises with MI [7,8]. The important gaps are clinical utility gaps. Which phenotype benefits? Which assay threshold is safe? Does cMyC reduce the observe zone in contemporary hs-cTn pathways [11,13]? Does it improve decision-making in CKD or HF, or does it simply add another abnormal result [15,21]? Does it identify a post-infarction risk phenotype that can be treated [14,32]? These gaps should be converted into phenotype-specific, endpoint-driven studies rather than addressed through additional descriptive biomarker cohorts. Table 4 summarizes the main evidence gaps, the trial designs required to address them, the populations in which they should be tested, and the minimum analytical requirements needed for interpretable results. This matrix emphasizes that each proposed cMyC use case requires a different evidentiary pathway. Early-presenter diagnosis, CKD/HF interpretation, suspected reinfarction, STEMI reperfusion phenotyping and prehospital or POCT deployment should not be validated with the same generic ACS cohort. Each requires phenotype-specific enrollment, predefined decision thresholds, clinically meaningful endpoints and assay-characterization standards appropriate to the intended use.

The ideal next studies are not small single-center diagnostic comparisons. They are prospective multicenter, platform-specific, decision-embedded trials [49,50]. A useful design would randomize EDs or patients to standard hs-cTn pathway versus hs-cTn plus cMyC pathway, with predefined discharge, observation, and rule-in criteria [12,53,54]. Outcomes should include missed MI, 30-day MACE, ED length of stay, hospitalization, downstream testing, cost, patient anxiety, and clinician adherence [50,52].

For prognosis, trials should define the action attached to a high cMyC value [14,40]. Intensified follow-up, early echocardiography, HF prevention clinic, CMR selection, or prolonged monitoring should be specified. Biomarker prognostication without an action plan rarely changes outcomes [49].

## 16. Limitations

This review is narrative and does not include a formal systematic-review risk-of-bias assessment, a GRADE certainty rating, or meta-analysis. The available evidence is heterogeneous with respect to assay platform, antibody epitopes, calibrators, sample matrix, patient selection, symptom-onset distribution, comparator hs-cTn assay, endpoint adjudication, and outcome definitions. Many pivotal diagnostic studies derive from overlapping investigative networks and related cohorts, which strengthens programmatic consistency but limits the independence of replication [7,11,30,31]. Several studies used pre-commercial or investigational assays, and independent external validation remains sparse [10,14,29,30]. The literature may also be affected by publication and selective-reporting bias and by financial, patent, or industry relationships within biomarker-development programs, even when these do not constitute conflicts of interest for the authors of this review. Cross-study comparisons, threshold transfer, and claims of clinical superiority should therefore be interpreted cautiously.

## 17. Conclusions

cMyC is a biologically plausible, analytically maturing, and clinically interesting sarcomere-derived biomarker signal whose most credible role is decision enhancement in selected ACS phenotypes. The most credible candidate use cases for further evaluation are early NSTEMI assessment and accelerated triage, including selected single-sample, 0/1 h, and dual-marker strategies; however, external validation has not consistently reproduced rule-out safety across platforms and populations. Prehospital testing, suspected reinfarction, and post-infarction risk stratification remain promising but require further phenotype-specific validation. The weakest claim is that cMyC should replace hs-cTnT or hs-cTnI testing. The literature does not justify that conclusion, and making it would reduce credibility. The field should instead pursue a more methodologically demanding and clinically meaningful question: where does cMyC produce decision-grade evidence beyond hs-cTnT, hs-cTnI, ECG, symptom timing, and clinical risk?

As an author-proposed translational roadmap, short-term priorities over the next 1–3 years should include assay, calibrator, and antibody-epitope harmonization; establishment of platform-specific reference intervals and clinically appropriate decision thresholds; complete and standardized study-level reporting; independent external validation of single-sample and 0/1 h algorithms; prespecified analyses in very early presenters and clinically relevant confounding phenotypes, including CKD, HF, and inherited cardiomyopathy; and improved characterization of circulating molecular forms and analytical transferability across platforms. Medium-to-long-term priorities over the subsequent 3–10 years should include randomized or cluster-randomized decision-embedded implementation trials; validation of point-of-care and prehospital pathways; demonstration of incremental clinical utility beyond contemporary hs-cTnT- and hs-cTnI-based algorithms; cost-effectiveness, workflow, and health-system impact analyses; outcome-guided post-infarction applications in which elevated cMyC is linked to a predefined management strategy; international analytical and clinical harmonization; and, only after reproducible patient-level benefit and safety have been demonstrated, consideration of incorporation into professional guidelines. These intervals represent an author-proposed translational development framework rather than evidence-based deadlines and are intended to distinguish the foundational analytical and external-validation work from the later implementation evidence required for clinical adoption.

If future trials demonstrate that cMyC safely increases immediate rule-out, reduces observation-zone burden, identifies high-risk post-infarction phenotypes, and is cost-effective across platforms and populations, it could become a meaningful adjunct in ACS pathways. Until then, cMyC remains a strong translational candidate whose clinical future depends not on molecular plausibility but on demonstrated patient-level benefit.

## Figures and Tables

**Table 3:** Phenotype-specific clinical utility and interpretive risk.

Phenotype	Expected Incremental Clinical Value	Why the Value May Be Real	Why It May Fail	Trial Endpoint That Matters
<1 h from pain onset [7,11,30,31]	Potentially high biological value; clinical safety unproven	Rapid release provides a biological rationale, but safe single-sample rule-out has not been established in <1 h presenters	Missed MI risk is high; symptom time often unreliable	30-day death/MI after early discharge; observation-zone reduction
1–3 h [7,11,30]	Moderate-high	Incremental value shown in early-onset analyses	hs-cTn deltas increasingly informative	Safe rule-out rate and ED length of stay
3–6 h [7,11,30]	Moderate	May increase triage efficacy or risk phenotype	Diagnostic acceleration advantage narrows	Disposition change without safety penalty
Late hs-cTn-positive NSTEMI [7,11,22]	Low diagnostic value	Could quantify injury burden	Diagnosis already evident	Prognosis or infarct-burden endpoint, not diagnosis
Suspected reinfarction [8,9]	Biologically plausible but unsupported by direct diagnostic-validation evidence	Faster clearance could theoretically help distinguish new injury from persistent post-infarction biomarker elevation	Adjudication is hard; evidence sparse	Recurrent MI accuracy with angiographic/imaging adjudication
CKD [15,20,43]	Uncertain	cMyC may show a different baseline distribution and dialysis-related behavior from hs-cTnT and hs-cTnI	Chronic injury and clearance confound specificity	Type 1 MI specificity, net benefit, and safety
HF/LV remodeling [15,21]	Prognostic rather than diagnostic	Reflects injury burden, hypertrophy, fibrosis, ventricular stress	Does not establish coronary mechanism	HF hospitalization, mortality, and management change
Women [29,44,45]	Potentially important	Sex-specific thresholds may reduce sex-related diagnostic misclassification	Unvalidated thresholds risk under/over-diagnosis	Sex-stratified sensitivity, specificity, NPV, PPV, and diagnostic reclassification
Type 2 MI/critical illness [22]	Limited etiologic value	Sensitive injury signal	Trigger and mechanism remain clinical	Management-changing classification and outcomes
STEMI after PCI [32,35,36,37,38]	Prognostic/quantitative	May track infarct size or reperfusion injury	hs-cTnI and CMR remain strong comparators	MVO, final infarct size, LV remodeling
Known or suspected inherited cardiomyopathy/pathogenic MYBPC3 variant [17,46,47]	Uncertain; chronic elevation is biologically plausible in manifest disease but has not been defined in genotype-positive/phenotype-negative carriers	Manifest MYBPC3-related hypertrophic cardiomyopathy may involve ventricular hypertrophy and adverse cardiac remodeling that could shift baseline cMyC distributions	Genotype-positive/phenotype-negative carriers are not equivalent to patients with manifest hypertrophic cardiomyopathy; baseline cMyC distributions and ED prevalence are unknown	Genotype- and phenotype-stratified reference distributions, acute delta performance, and specificity for type 1 MI

Abbreviations: CKD, chronic kidney disease; cMyC, circulating cMyBP-C biomarker signal; CMR, cardiac magnetic resonance; ED, emergency department; HF, heart failure; hs-cTn, cardiac troponin measured with a high-sensitivity assay; hs-cTnI, cardiac troponin I measured with a high-sensitivity assay; hs-cTnT, cardiac troponin T measured with a high-sensitivity assay; LV, left ventricular; MI, myocardial infarction; MVO, microvascular obstruction; NPV, negative predictive value; PCI, percutaneous coronary intervention; PPV, positive predictive value; STEMI, ST-segment elevation myocardial infarction.

## Data Availability

No new data were created or analyzed in this study. Data sharing is not applicable to this article.

## Supplementary Materials

The following supporting information can be downloaded at: https://www.mdpi.com/article/10.3390/ijms27156934/s1.

## Abbreviations

The following abbreviations are used in this manuscript:

ACS	acute coronary syndrome
APACE	Advantageous Predictors of Acute Coronary Syndrome Evaluation
AUC	area under the receiver operating characteristic curve
CI	confidence interval
AMI	acute myocardial infarction
CKD	chronic kidney disease
CMR	cardiac magnetic resonance
cMyBP-C	cardiac myosin-binding protein C
cMyC	circulating cMyBP-C biomarker signal
cTnI	cardiac troponin I
cTnT	cardiac troponin T
CRP	C-reactive protein
ECG	electrocardiogram
ED	emergency department
eGFR	estimated glomerular filtration rate
ELISA	enzyme-linked immunosorbent assay
ESC	European Society of Cardiology
HF	heart failure
GRADE	Grading of Recommendations Assessment, Development and Evaluation
hs-cTn	cardiac troponin measured with a high-sensitivity assay
hs-cTnI	cardiac troponin I measured with a high-sensitivity assay
hs-cTnT	cardiac troponin T measured with a high-sensitivity assay
LoD	limit of detection
LoQ	limit of quantification
LV	left ventricular
MACE	major adverse cardiovascular events
MI	myocardial infarction
MVO	microvascular obstruction
MYBPC3	myosin-binding protein C3 gene
MYC/c-Myc	MYC proto-oncogene/protein c-Myc
NPV	negative predictive value
NR	not reported
NSTEMI	non-ST-segment elevation myocardial infarction
NT-proBNP	N-terminal pro-B-type natriuretic peptide
PCI	percutaneous coronary intervention
POCT	point-of-care testing
PPV	positive predictive value
STEMI	ST-segment elevation myocardial infarction
TAT	turnaround time
