# Supplementary material for: Cardiac Myosin-Binding Protein C in Suspected Acute Coronary Syndrome: From Sarcomeric Injury Biology to Decision-Grade Risk Stratification
Source: Int J Mol Sci. 2026 Aug 2;27(15):6934. doi: 10.3390/ijms27156934 (PMC13467487; doi:10.3390/ijms27156934)
Supplement: Supplementary file 1 [file ijms-27-06934-s001.zip › ijms-4449901-supplementary.pdf]

Supplementary Tables

Cardiac Myosin-Binding Protein C in Suspected Acute Coronary Syndrome: From Sarcomeric Injury Biology to Decision-Grade Risk Stratification

Abbreviations: *AHF, acute heart failure; AMI, acute myocardial infarction; APACE, Advantageous Predictors of Acute Coronary Syndrome Evaluation; ACS, acute coronary syndrome; AUC, area under the receiver operating characteristic curve; CABG, coronary artery bypass grafting; CAD, coronary artery disease; CI, confidence interval; CKD, chronic kidney disease; CLSI, Clinical and Laboratory Standards Institute; CMR, cardiac magnetic resonance; cMyBP-C, cardiac myosin-binding protein C; cMyC, circulating cMyBP-C biomarker signal; CV, coefficient of variation; CVA, analytical coefficient of variation; CVI, within-subject biological coefficient of variation; CVG, between-subject biological coefficient of variation; ED, emergency department; eGFR, estimated glomerular filtration rate; ELISA, enzyme-linked immunosorbent assay; ESC, European Society of Cardiology; GRADE, Grading of Recommendations Assessment, Development and Evaluation; HF, heart failure; HISCL, high-sensitivity chemiluminescence enzyme immunoassay; HR, hazard ratio; hs-cTnI, cardiac troponin I measured with a high-sensitivity assay; hs-cTnT, cardiac troponin T measured with a high-sensitivity assay; LoD, limit of detection; LoQ, limit of quantification; LV, left ventricular; MACE, major adverse cardiovascular events; MI, myocardial infarction; MVO, microvascular obstruction; NPV, negative predictive value; NR, not reported; NSTEMI, non-ST-segment elevation myocardial infarction; NT-proBNP, N-terminal pro-B-type natriuretic peptide; PCI, percutaneous coronary intervention; POCT, point-of-care testing; PPV, positive predictive value; RCV, reference change value; STEMI, ST-segment elevation myocardial infarction; TASH, transcoronary ablation of septal hypertrophy; ULOQ, upper limit of quantification.*

Table S1. Study-level evidence matrix and qualitative assessment of heterogeneity.

Study / reference	Design and population	cMyC assay / platform	Sampling window	Intended use	Reported key metrics / thresholds	Renal or phenotype handling	Missing or heterogeneity issue	Interpretive confidence
**Kuster et al., 2014 [8]**	Porcine cardiac injury model plus clinical cohorts undergoing transcoronary ablation of septal hypertrophy and patients with NSTEMI	Custom electrochemiluminescence cMyBP-C immunoassay targeting the cardiac-specific C0 domain	Serial controlled-injury sampling; clinical sampling varied by cohort	Release kinetics and proof of circulating detectability	Earlier rise and a different overall release-clearance profile than cardiac troponin in controlled injury models; ED AUC, NPV and specificity: NR	Hypertrophic cardiomyopathy represented in the ablation cohort; renal-function analysis: NR	Mixed experimental and clinical populations; not designed to establish ED cut-offs	Moderate for release kinetics; limited for ED threshold transfer
**Baker et al., 2015 [9]**	Small prospective serial cohorts with STEMI, transcoronary ablation of septal hypertrophy, and CABG	Research assays evaluating full-length cMyBP-C and circulating fragments	Serial samples after injury or reperfusion	Early kinetics and molecular processing	The circulating cMyC signal peaked earlier than hs-cTnT in the STEMI cohort (9.3 ± 3.1 vs 11.8 ± 3.4 h; $p<0.007$) and the TASH cohort (9.7 ± 1.4 vs 21.6 ± 1.4 h; $p<0.0001$), and showed faster post-CABG clearance (decay half-time 5.5 ± 0.8 vs 22 ± 5 h; $p<0.0001$). Diagnostic AUC, NPV, and specificity: NR	Renal stratification: NR; controlled procedural cohorts differ from undifferentiated ED patients	Small cohorts, procedure-specific injury, and assay-dependent molecular forms	Moderate for mechanistic kinetics; limited for universal clinical cut-offs

Study / reference	Design and population	cMyC assay / platform	Sampling window	Intended use	Reported key metrics / thresholds	Renal or phenotype handling	Missing or heterogeneity issue	Interpretive confidence
**Marjot et al., 2016 [10]**	Analytical development and application of a high-sensitivity immunoassay	Singulex Erenna high-sensitivity immunoassay using the N-terminal 1A4/3H8 antibody pair	Analytical/reference-population sampling; clinical timing varied by application cohort	Analytical validation and reference limit	LoD 0.4 ng/L; LoQ 1.2 ng/L; observed 99th percentile 87 ng/L in a selected clinically stable coronary-angiography cohort without obstructive coronary artery disease	Dedicated CKD or HF diagnostic strata: NR	The 87 ng/L value was not derived from a CLSI-aligned healthy reference population and is not an optimized ACS rule-out or rule-in threshold	High for the reported LoD, LoQ, and assay-development characteristics; limited for treating 87 ng/L as a general healthy upper reference limit or ACS threshold
**Kaier et al., 2017 [7]**	Prospective multicenter cohort of 1,954 patients with suspected AMI; direct comparison with hs-cTnT and hs-cTnI	High-sensitivity Erenna cMyC assay	Presentation sampling with analyses by symptom timing, including <3 h	Early AMI diagnosis and single-sample triage	AUC 0.924; reported presentation rule-out threshold ≤10 ng/L and rule-in threshold >120 ng/L	No dedicated genotype-informed analysis; renal-function effects not the primary comparison	Thresholds were derived within the studied diagnostic program and are platform-specific; symptom-onset distribution and comparator assays affect performance.	Moderate-to-high for diagnostic discrimination and internally derived thresholds; external platform and population validation required.
**Kaier et al., 2019 [31]**	Prospective prehospital diagnostic cohort of 776 patients with retrospective blinded quantification of cMyC and hs-cTnT in stored ambulance-obtained samples, including a very-early subgroup	Laboratory high-sensitivity cMyC assay applied to prehospital samples	Prehospital presentation; subgroup <60 min; later timing strata also assessed	Prehospital biochemical triage	AUC 0.839; sensitivity and NPV reached 100% at the 10 ng/L threshold after at least 2 h of symptoms in the reported cohort	Renal/heart-failure subgroup performance: NR	Laboratory assay rather than definitive bedside POCT; performance depends strongly on symptom timing	Moderate; clinically relevant setting but requires external POCT and safety validation

Study / reference	Design and population	cMyC assay / platform	Sampling window	Intended use	Reported key metrics / thresholds	Renal or phenotype handling	Missing or heterogeneity issue	Interpretive confidence
**Kaier et al., 2022 [30]**	Prospective derivation and validation of a cMyC 0/1 h algorithm; validation cohort n=663	High-sensitivity cMyC platform linked to prior Erenna development	0 h and 1 h serial sampling	Rule-out, observe-zone and rule-in allocation	Rule-out: 0 h <10 ng/L, or 0 h <18 ng/L with 1 h delta <4 ng/L; rule-in: 0 h ≥140 ng/L or delta ≥15 ng/L. Validation NPV 99.6% (95% CI 98.9–100), sensitivity 99.1% (95% CI 97.0–100), specificity 93.6% (95% CI 91.4–95.6), and PPV 71.1% (95% CI 63.1–79.0).	CKD/HF were present within real-world cohorts but not sufficient to establish phenotype-specific cut-offs	Internal algorithm validation does not establish interchangeability with other platforms or populations. The study was derived from the APACE program; participant overlap with subsequent APACE analyses, including Lopez-Ayala et al. [11], is possible, and the reports should not be treated as fully independent evidence.	High for the prespecified algorithm within the reported Erenna platform and validation cohort; external platform, population, and phenotype validation remains required.

Study / reference	Design and population	cMyC assay / platform	Sampling window	Intended use	Reported key metrics / thresholds	Renal or phenotype handling	Missing or heterogeneity issue	Interpretive confidence
**Lopez-Ayala et al., 2025 [11]**	Secondary analysis of an international prospective ED cohort of 4,735 patients with suspected NSTEMI, including 854 patients with adjudicated NSTEMI; findings externally validated in an independent international cohort	Novel prototype automated cMyC immunoassay on a fully automated laboratory platform, integrated with contemporary hs-cTnT pathways	cMyC, hs-cTnT and hs-cTnI measured upon ED presentation; single-blood-draw dual-biomarker strategy	Dual-marker triage and observe-zone reduction	At presentation, cMyC AUC 0.943 (95% CI 0.936–0.950) vs hs-cTnT 0.936 (95% CI 0.929–0.944; p=0.008); in patients presenting within ≤3 h, 0.939 (95% CI 0.928–0.951) vs 0.921 (95% CI 0.907–0.936; p<0.001). Triage efficacy increased from 26.8% to 60.0% with the dual-biomarker strategy without compromising reported index-visit safety	Dedicated renal, HF and inherited-cardiomyopathy thresholds: NR	Incremental diagnostic performance is not equivalent to randomized implementation benefit. The primary analysis arose from the APACE study program and may partially overlap with Kaier et al. [30]. The independent external validation cohort should be distinguished from the potentially overlapping APACE dataset.	Moderate-to-high for incremental diagnostic evidence; limited for analytical transferability; randomized clinical-utility testing remains required.

Study / reference	Design and population	cMyC assay / platform	Sampling window	Intended use	Reported key metrics / thresholds	Renal or phenotype handling	Missing or heterogeneity issue	Interpretive confidence
**Yildirim et al., 2025 [13]**	Single-center observational analysis of 1,765 emergency-department patients with suspected NSTEMI, including 212 patients with adjudicated NSTEMI	Research cMyC measurement combined with hs-cTnT and copeptin	Presentation/accelerated serial pathways as reported	Rapid rule-out using cMyC alone or in dual-marker combinations	Dual-marker NPV 99.1-100% and sensitivity 96.2-100%, depending on the marker combination and threshold applied in the reported analyses	Phenotype-specific renal and HF thresholds: NR	Multiple marker combinations and pathway definitions limit direct comparison with other studies. This analysis used the same 1,765-patient Heidelberg cohort as Yildirim et al. [32]; the two publications represent complementary analyses of the same study population and should not be interpreted as independent validation cohorts.	Moderate; requires external validation and decision-embedded testing
**Hotchi et al., 2026 [29]**	Prospective Japanese suspected-ACS cohort within 12 h of symptom onset plus reference-limit analysis	Automated Sysmex HISCL cMyC platform	0 h/1 h diagnostic sampling; reference-population sampling	Platform-specific 0/1 h validation and sex-specific reference limits	cMyC rule-out: 43.8% at 0 h and 56.5% after 1 h; NPV 95.2%, sensitivity 83.3%. cMyC rule-in: 10.0% at 0 h and 19.2% after 1 h; PPV 70.0%, specificity 90.3%. hs-cTnT: rule-out 48.8%, NPV 100%, sensitivity 100%; rule-in 25.4%, PPV 62.1%, specificity 83.6%. Sex-specific 99th-percentile concentrations: 11.4 ng/L in men and 8.1 ng/L in women.	Population-specific analysis performed; dedicated CKD/hemodialysis thresholds: NR	Rule-out safety was substantially lower for cMyC than for hs-cTnT; the small single-country cohort and distinct HISCL platform limit transferability.	Moderate for external platform validation; limited for stand-alone rule-out because cMyC sensitivity of 83.3% and NPV of 95.2% were insufficient for discharge.

Study / reference	Design and population	cMyC assay / platform	Sampling window	Intended use	Reported key metrics / thresholds	Renal or phenotype handling	Missing or heterogeneity issue	Interpretive confidence
**Hasselbalch et al., 2024 [20]**	Cohort study in 307 patients receiving hemodialysis	cMyC research assay; platform characteristics should be taken from the original report	Baseline and dialysis-related sampling as reported	Effect of hemodialysis and renal failure on myocardial-injury biomarkers	Before dialysis, hs-cTnT exceeded the 99th percentile in 299 patients (99%), cMyC in 188 patients (66%), and hs-cTnI in 35 patients (11%). During dialysis, hs-cTnT decreased by 23% ($p<0.001$), hs-cTnI by 15% ($p=0.049$), and cMyC by 4% ($p=0.256$). cMyC was not associated with mortality or the combined cardiovascular-event/mortality endpoint.	Dedicated hemodialysis population	Chronic injury and dialysis timing limit transfer of general-population cut-offs; the absence of a prognostic association for cMyC should not be interpreted as evidence of diagnostic specificity.	Moderate-to-high for the reported dialysis-related biomarker distributions and prognostic associations; limited for AMI diagnostic thresholds
**Kozhuharov et al., 2021 [21]**	Prospective multicenter acute-dyspnoea/acute-heart-failure cohort; diagnostic analysis n=1,083 (biomarker AUC comparison n=1,060)	High-sensitivity cMyC research assay	Acute-presentation and follow-up sampling as reported	Diagnosis and risk stratification in acute HF	AHF diagnosis: cMyC AUC 0.81 (95% CI 0.78–0.83), hs-cTnT AUC 0.79 (95% CI 0.76–0.82), and NT-proBNP AUC 0.91. At cMyC 16 ng/L: sensitivity 95% (95% CI 93–97), specificity 37% (95% CI 33–41), NPV 88% (95% CI 84–92), PPV 61% (95% CI 57–64), with 21% ruled out.	Dedicated acute-HF population	The population presented with acute dyspnoea/AHF rather than suspected ACS; diagnostic performance for AHF does not establish type 1 MI specificity.	Moderate-to-high for the reported AHF diagnostic performance; moderate for HF prognostic associations; limited for ACS/type 1 MI interpretation

Study / reference	Design and population	cMyC assay / platform	Sampling window	Intended use	Reported key metrics / thresholds	Renal or phenotype handling	Missing or heterogeneity issue	Interpretive confidence
**Rajakulasingam et al., 2026 [32]**	Reperfused STEMI cohort with serial cMyC and imaging-linked evaluation	In-house electrochemiluminescence sandwich ELISA using high-affinity N-terminal capture and detection monoclonal antibodies; serum; LoD 14 ng/L at a CV of 12%, with a reported detection range up to 80,000 ng/L	Serial post-PCI/reperfusion sampling	Infarct burden, microvascular obstruction and remodeling phenotype	CMR-linked associations; diagnostic AUC/NPV not applicable because STEMI diagnosis was established	Renal/genetic stratification: NR	Conceptual prognostic application; requires external validation and management-changing endpoints	Moderate for biological association; limited for clinical utility
**Chen et al., 2026 [14]**	Prospective single-center cohort of 285 patients with AMI presenting within 12 h of symptom onset; 30-day MACE incidence 27.4%	Investigational gold-nanocage-based colloidal-gold POCT (Nanjing Bottests) using monoclonal anti-cMyC antibodies	Single admission plasma sample; result available in approximately 15 min	Early prognostic risk stratification after established AMI	Reported analytical detection sensitivity 0.1 pg/mL; adjusted HR for 30-day MACE per unit increase in natural-log-transformed cMyC 1.322 (95% CI 1.090-1.603; p=0.005); exploratory cut-offs 220 and 487 pg/mL	Subgroup analyses included renal function and MI type; patients with uremia or hypertrophic cardiomyopathy were excluded	Single-center established-AMI cohort, single-time-point measurement, and percentile/data-driven thresholds not validated for ACS diagnosis	Moderate for prognostic association and operational POCT feasibility; limited for incremental prediction and diagnostic threshold transfer
**Paus et al., 2025 [15]**	Prospective population-based ACE 1950 cohort; n=3,672, with CMR substudy n=199	High-sensitivity Millipore Sigma Erenna assay	Single baseline fasting venous blood sample with echocardiographic and CMR phenotyping	Chronic myocardial injury, cardiac remodeling, and population determinants	cMyC measurable in 99.6%; associated with LV mass, left atrial volume, renal function, systolic/diastolic function, and focal fibrosis; diagnostic AUC, NPV, and specificity: NR	General-population cohort with multivariable renal and structural-cardiac phenotyping	Age-cohort population born in 1950; not designed for ACS diagnosis or acute clinical thresholds	High for population associations; limited for acute diagnostic interpretation

Study / reference	Design and population	cMyC assay / platform	Sampling window	Intended use	Reported key metrics / thresholds	Renal or phenotype handling	Missing or heterogeneity issue	Interpretive confidence
**Alaour et al., 2022 [33]**	Multicenter biological-variation study in 30 healthy volunteers with weekly sampling for 10 weeks	Erenna high-sensitivity cMyC assay with duplicate measurements	Non-fasting serum samples obtained once weekly for 10 consecutive weeks	Within- and between-subject variation and serial-change interpretation	Median 4.38 ng/L; CVA 19.5%, CVI 17.8%, CVG 66.9%; positive RCV +106.7%, negative RCV -51.6%; index of individuality 0.42	eGFR <60 mL/min/1.73 m2 and known cardiac disease were exclusion criteria	Small, relatively young healthy cohort; RCV was not validated in ACS, CKD, HF, or older populations	High for the reported biological-variation estimates; limited for patient-level acute delta thresholds
**Yildirim et al., 2025 [34]**	Retrospective ED registry analysis of 1,765 patients with suspected ACS; 212 NSTEMI cases	Research cMyC assay compared with hs-cTnT and a broad biomarker panel	Presentation biomarker panel with evaluation of ESC 0/1 h and 0/3 h pathways	Comparative diagnostic discrimination and short- and long-term prognosis	cMyC AUC 0.917 vs hs-cTnT 0.922; ESC 0/1 h sensitivity/NPV 100%/100%; ESC 0/3 h 96.8%/99.3%; 1-year event rates 0.0-3.4% across evaluated protocols	Renal- and HF-specific cMyC thresholds: NR	Retrospective single-center design; comparative biomarker analysis did not establish a stand-alone cMyC decision threshold. This analysis used the same 1,765-patient Heidelberg cohort as Yildirim et al. [13]; the two publications represent complementary analyses of the same study population and should not be interpreted as independent validation cohorts.	Moderate for comparative discrimination; limited for implementation and threshold transfer

Study / reference	Design and population	cMyC assay / platform	Sampling window	Intended use	Reported key metrics / thresholds	Renal or phenotype handling	Missing or heterogeneity issue	Interpretive confidence
**Shen et al., 2025 [39]**	Single-center angiography cohort n=367 plus prospective 1-year follow-up of 190 patients with stable CAD	Commercial colloidal-gold immunochromatographic analyzer (Botian Kezhi); detection range 0-5,000 pg/mL; interassay CV <15%	Fasting arterial plasma obtained during cardiac catheterization	Stable-CAD presence, angiographic severity, and 1-year prognosis	CAD AUC 0.827, cut-off 19.5 pg/mL (70.3% sensitivity, 81.4% specificity); severe CAD AUC 0.778, cut-off 23.5 pg/mL (80.2%/68.1%); >=27 pg/mL associated with 1-year MACE	eGFR included in multivariable analysis; patients with acute ACS were excluded	Stable-CAD population and device-specific pg/mL scale; thresholds are not ACS rule-out/rule-in cut-offs and require external validation	Moderate for stable-CAD association; limited for ACS or cross-platform interpretation

Note: NR denotes not reported or not unambiguously extractable from the study report. Interpretive confidence is a narrative, context-specific assessment and is not a formal GRADE rating. High confidence was assigned when a study was prospective, included multicenter recruitment or external validation, used prespecified thresholds, reported the principal diagnostic metrics and sampling windows, and adequately described the assay. Moderate confidence was assigned when one or two of these elements were incomplete but the study remained interpretable for its intended context. Limited confidence was assigned when major diagnostic metrics or sampling windows were missing, thresholds were predominantly post hoc, assay characterization was insufficient, or the design did not permit transfer to the proposed clinical use. For non-diagnostic analytical or mechanistic studies, confidence was restricted to the directly reported analytical or mechanistic claim and did not imply clinical validation. No numeric score was calculated. Reference numbers correspond to the main manuscript.

Table S2. Assay architecture, analytical characteristics, reference limits, and reported clinical thresholds.

Study / reference	Platform / format	Antibodies or epitope region	Molecular species recognized	Matrix	LoD	LoQ	Reference interval / 99th percentile	Reported clinical threshold(s)	Transferability note
**Kuster et al., 2014 [8]**	Custom electrochemiluminescence immunoassay used in controlled-injury and clinical cohorts	Capture and detection antibodies directed against the cardiac-specific C0 domain	Circulating cMyC signal comprising intact cMyBP-C and/or injury-related fragments; exact intact-versus-fragment composition was not resolved	Serum/plasma as reported by cohort	116 ng/L	640 ng/L	NR	No universal ACS cut-off established	The reported analytical range and calibration differ substantially from later high-sensitivity Erenna assays; concentrations should not be considered interchangeable. The reported upper limit of quantification was 400,000 ng/L.
**Baker et al., 2015 [9]**	Research immunoassays for intact protein and fragments	Multiple antibodies targeting cMyBP-C regions in mechanistic analyses	Full-length cMyBP-C and circulating fragments were evaluated, but clinical assays did not isolate every individual isoform	Serum	NR	NR	NR	No validated ED rule-out/rule-in threshold	Useful for fragment biology and kinetics; not a standardized clinical platform
**Marjot et al., 2016 [10]**	Singulex Erenna high-sensitivity immunoassay	1A4 and 3H8 monoclonal antibodies directed against cardiac-restricted N-terminal epitopes within the C0–C2 construct	May detect intact cMyBP-C together with N-terminal-containing fragments; not demonstrated to quantify one isolated fragment isoform	Serum	0.4 ng/L	1.2 ng/L	Observed 99th percentile of the selected clinically stable angiography-cohort distribution: 87 ng/L; the study did not establish a healthy-population 99th-percentile upper reference limit	Analytical development study; clinical thresholds were derived in subsequent studies	Platform-defining analytical study; values are assay- and calibrator-dependent

Study / reference	Platform / format	Antibodies or epitope region	Molecular species recognized	Matrix	LoD	LoQ	Reference interval / 99th percentile	Reported clinical threshold(s)	Transferability note
**Kaier et al., 2017 [7]**	Singulex Erenna high-sensitivity cMyC assay	N-terminal 1A4/3H8 epitope pair inherited from the assay-development platform	Likely mixed N-terminal-epitope-containing circulating species; exact molecular-species composition was not directly established in the clinical study	Serum	As validated for the platform [10]	As validated for the platform [10]	99th percentile inherited from platform validation; not used as a universal diagnostic cut-off	Presentation rule-out ≤10 ng/L; rule-in >120 ng/L	Thresholds apply to the studied platform, population and context of use
**Kaier et al., 2019 [31]**	Laboratory high-sensitivity cMyC assay applied to prehospital samples	N-terminal high-sensitivity assay configuration	Likely mixed N-terminal-epitope-containing circulating species; exact molecular-species composition was not directly established in the clinical study	Stored ambulance-obtained blood samples; exact matrix and anticoagulant were not unambiguously reported in the main article (NR)	0.4 ng/L for the underlying Erenna assay [10]	1.2 ng/L for the underlying Erenna assay [10]	NR in the original article	10 ng/L evaluated for rule-out; sensitivity and NPV reached 100% after at least 2 h of symptoms in the reported cohort	Prehospital collection does not establish bedside POCT equivalence; timing-specific safety requires prospective validation

Study / reference	Platform / format	Antibodies or epitope region	Molecular species recognized	Matrix	LoD	LoQ	Reference interval / 99th percentile	Reported clinical threshold(s)	Transferability note
**Kaier et al., 2022 [30]**	Pre-commercial high-sensitivity Singulex Erenna cMyC assay for a 0/1 h algorithm	N-terminal high-sensitivity assay configuration	Likely mixed N-terminal-epitope-containing circulating species; exact molecular-species composition was not directly established in the clinical study	Heparin plasma and serum samples collected at presentation and serially	0.4 ng/L	1.2 ng/L	No universal reference limit used as the diagnostic decision threshold	Rule-out: 0 h <10 ng/L, or 0 h <18 ng/L with 1 h delta <4 ng/L; rule-in: 0 h >=140 ng/L or 1 h delta >=15 ng/L	Thresholds are specific to the Erenna platform, studied population, and sampling interval; analytical bridging is required before transfer
**Lopez-Ayala et al., 2025 [11]**	Novel prototype automated cMyC immunoassay on a fully automated laboratory platform	Antibody and epitope configuration not fully reported in the clinical article	Assay-dependent circulating cMyC signal; exact intact-versus-fragment composition was not established	Exact matrix was not unambiguously reported in the accessible article record (NR)	NR in the original article record available to this review	NR in the original article record available to this review	NR	Exact cMyC decision cut-offs were not unambiguously extractable from the accessible article record (NR); reported dual-marker triage efficacy was 60.0% versus 26.8% with hs-cTnT alone	Incremental performance is pathway-specific and does not establish a transferable stand-alone cMyC threshold
**Hotchi et al., 2026 [29]**	Automated Sysmex HISCL cMyC platform	Platform-specific antibodies; detailed epitope mapping was not reported in the accessible article record	Assay-dependent cMyBP-C forms; intact/fragment specificity not established	Exact matrix was not unambiguously reported in the accessible article record (NR)	NR in the original article record available to this review	NR in the original article record available to this review	Sex-specific 99th-percentile concentrations: 11.4 ng/L in men and 8.1 ng/L in women	The previously published cMyC 0/1 h algorithm was evaluated; exact platform-specific decision cut-offs were not unambiguously extractable from the accessible article record (NR)	Direct transfer of Erenna-derived concentrations to the HISCL platform should not be assumed; external validation showed insufficient rule-out sensitivity and NPV for stand-alone discharge.

Study / reference	Platform / format	Antibodies or epitope region	Molecular species recognized	Matrix	LoD	LoQ	Reference interval / 99th percentile	Reported clinical threshold(s)	Transferability note
**Hasselbalch et al., 2024 [20]**	Established high-sensitivity cMyC assay on the Singulex Erenna platform; samples assayed in duplicate	N-terminal high-sensitivity assay configuration; exact antibody pair was not restated in the clinical article	Likely mixed N-terminal-epitope-containing circulating species; exact molecular-species composition was not directly established	Pre- and post-dialysis blood samples; exact matrix was not unambiguously reported in the accessible article record (NR)	0.4 ng/L for the underlying Erenna assay [10]	1.2 ng/L for the underlying Erenna assay [10]	99th-percentile benchmark of 87 ng/L derived from assay development [10]; not established in the hemodialysis cohort	No ACS diagnostic cut-off; 66% of patients exceeded the referenced 99th percentile before dialysis	Dedicated hemodialysis population; chronic myocardial injury and dialysis-related kinetics prevent transfer of general-population ACS thresholds
**Kozhuharov et al., 2021 [21]**	Established high-sensitivity cMyC assay on the Erenna platform, performed by Millipore Sigma	N-terminal C0-region detection configuration; exact antibody pair was not restated in the clinical article	Likely mixed N-terminal-epitope-containing circulating species; exact molecular-species composition was not directly established	Plasma	0.4 ng/L for the underlying Erenna assay [10]	1.2 ng/L for the underlying Erenna assay [10]	99th-percentile benchmark of 87 ng/L derived from assay development [10]; not newly established in this acute-heart-failure cohort	16 ng/L for rapid exclusion of acute heart failure; median 72 ng/L used for prognostic stratification. These are not ACS rule-out/rule-in thresholds	Acute-dyspnoea/acute-heart-failure population; diagnostic and prognostic thresholds should not be transferred to suspected ACS or type 1 MI

Study / reference	Platform / format	Antibodies or epitope region	Molecular species recognized	Matrix	LoD	LoQ	Reference interval / 99th percentile	Reported clinical threshold(s)	Transferability note
**Rajakulasingam et al., 2026 [34]**	In-house electrochemiluminescence sandwich ELISA; duplicate batch analysis	High-affinity N-terminal capture and detection monoclonal antibodies	Assay-dependent N-terminal-containing cMyBP-C forms; exact intact-versus-fragment specificity was not established	Serum	14 ng/L at CV 12%	NR	99th-percentile upper reference limit 87 ng/L cited from prior assay development; not newly established in this STEMI cohort	Exploratory MVO thresholds: 6 h >2,388 ng/L, 12 h >1,275 ng/L, 18 h >741 ng/L, and 24 h >835 ng/L; these are not ACS diagnostic rule-out/rule-in thresholds	Prototype non-commercial assay with a higher LoD and therefore lower analytical sensitivity than optimized Erenna platforms; post-reperfusion thresholds require external validation
**Chen et al., 2026 [14]**	Investigational gold-nanocage-based colloidal-gold POCT; reported turnaround time approximately 15 min	Monoclonal anti-cMyC antibodies; device-specific epitope locations: NR in the original article	Device-dependent circulating cMyC signal; molecular-species specificity was not established	Plasma	Reported analytical detection sensitivity 0.1 pg/mL; a formal CLSI LoD was not reported	NR in the original article	No reference interval or 99th-percentile upper reference limit established	Exploratory prognostic categories: <220, 220 to <487, and >=487 pg/mL; these were percentile/data-driven and not validated ACS diagnostic thresholds	Whole-blood performance was not established; analytical validation and bridging to central-laboratory assays remain required

Study / reference	Platform / format	Antibodies or epitope region	Molecular species recognized	Matrix	LoD	LoQ	Reference interval / 99th percentile	Reported clinical threshold(s)	Transferability note
**Yildirim et al., 2025 [13]**	Research high-sensitivity cMyC assay combined with Roche hs-cTnT and/or copeptin	N-terminal high-sensitivity assay configuration; exact epitope pair not restated in the clinical article	Likely mixed N-terminal-epitope-containing circulating species; exact molecular-species composition was not directly established in the clinical study	Exact matrix was not unambiguously reported in the accessible article record (NR)	Formal analytical LoD not separately reported in the clinical article	NR in the original article	NR	Single-marker cMyC thresholds <10 ng/L and <2.3 ng/L; dual-marker combinations were evaluated with hs-cTnT and copeptin (<10 pmol/L)	Threshold performance depended on the marker combination and clinical pathway; external validation is required
**Alaour et al., 2022 [33]**	Singulex Erenna high-sensitivity cMyC assay with duplicate measurements	1A4/3H8 N-terminal epitope configuration inherited from assay development [10]	Likely mixed N-terminal-epitope-containing circulating species; exact molecular-species composition was not directly established in the clinical study	Serum	0.4 ng/L	1.2 ng/L	Healthy-cohort median 4.38 ng/L; no 99th-percentile upper reference limit established in this study	Biological reference change values: +106.7% and -51.6%; these are not ACS diagnostic cut-offs	RCVs were derived in 30 healthy volunteers and should not be transferred directly to ACS, CKD, HF, or older populations

Study / reference	Platform / format	Antibodies or epitope region	Molecular species recognized	Matrix	LoD	LoQ	Reference interval / 99th percentile	Reported clinical threshold(s)	Transferability note
**Paus et al., 2025 [15]**	High-sensitivity Millipore Sigma Erenna assay	N-terminal high-sensitivity epitope configuration	Likely mixed N-terminal-epitope-containing circulating species; exact molecular-species composition was not directly established in the clinical study	Fasting venous blood samples; processed matrix (serum vs plasma) was not explicitly stated in the main article	0.4 ng/L	1.2 ng/L	No single universal upper reference limit was established. Study-derived overall 99th percentiles ranged from 27.1 to 115.8 ng/L across populations with differing risk-factor and imaging profiles; among apparently healthy participants, the 99th percentile was 17.8 ng/L in women and 27.5 ng/L in men. The previously reported 87 ng/L value derived from a selected stable angiography cohort [10]	No ACS rule-out/rule-in threshold; population-association study	General-population associations should not be interpreted as acute diagnostic thresholds; the assay ULOQ was 2,000 ng/L

Study / reference	Platform / format	Antibodies or epitope region	Molecular species recognized	Matrix	LoD	LoQ	Reference interval / 99th percentile	Reported clinical threshold(s)	Transferability note
**Yildirim et al., 2025 [34]**	Research cMyC assay compared with hs-cTnT and a broad biomarker panel	Exact antibody/epitope configuration was not restated in the clinical article	Assay-dependent circulating cMyC signal	Exact matrix was not unambiguously reported in the accessible article record (NR)	NR in the original article	NR in the original article	NR	No stand-alone cMyC rule-out or rule-in cut-off established; cMyC AUC was 0.917 in the comparative analysis	Comparative AUC performance does not establish a transferable diagnostic threshold or clinical utility
**Shen et al., 2025 [39]**	Commercial colloidal-gold immunochromatographic analyzer (Botian Kezhi); measurement range 0-5,000 pg/mL; interassay CV <15%	Device-specific antibodies and epitope locations: NR in the original article	Device-dependent circulating cMyC signal; intact-versus-fragment specificity was not established	Fasting arterial plasma	Formal LoD: NR in the original article	Formal LoQ: NR in the original article	No healthy reference interval or 99th-percentile upper reference limit established	Stable CAD: 19.5 pg/mL for CAD presence, 23.5 pg/mL for severe CAD, and >=27 pg/mL for prognostic stratification	These are stable-CAD, device-specific thresholds and must not be applied as ACS rule-out/rule-in cut-offs

Note: NR denotes not reported or not unambiguously extractable. N-terminal epitope-pair assays may recognize intact cMyBP-C and multiple N-terminal-containing fragments; the term fragment-specific should be used only when molecular-species specificity has been experimentally demonstrated. Concentrations, reference limits and cut-offs are platform- and context-dependent. Reference numbers correspond to the main manuscript.
